# Where are we going and where have we been? Examining the effects of maps on spatial learning in an indoor guided navigation task

**DOI:** 10.1186/s41235-020-00213-w

**Published:** 2020-03-20

**Authors:** Mallory C. Stites, Laura E. Matzen, Zoe N. Gastelum

**Affiliations:** Sandia National Laboratories, PO Box 5800, Albuquerque, NM 87185-0152 USA

**Keywords:** Spatial knowledge, Navigation, Maps, Sense of direction, Guided indoor navigation, Landmark knowledge, Survey knowledge, Intentional vs. incidental learning

## Abstract

**Background:**

International Atomic Energy Agency (IAEA) safeguards inspectors are faced with the difficult task of learning the layout of complex nuclear facilities while being escorted through the facilities. This study addresses a gap in the literature regarding how to best support the development of inspectors’ spatial knowledge, given the constraint that they cannot bring digital devices into most nuclear facilities. We tested whether viewing a map before learning a guided route or carrying a map along the route enabled better spatial learning than having no exposure to a map. Moreover, we tested the impact of carrying maps with different levels of detail (simple 2D, simple 3D, or complex 2D maps) on spatial learning outcomes, as well as interactions between map type and individual differences in sense of direction.

**Results:**

The results showed nearly opposite patterns of performance for participants with good and poor sense of direction scores. Participants with a good sense of direction showed higher levels of spatial knowledge when studying or carrying simple maps, whether 2D or 3D, but they did not benefit from using a complex map. Participants with a poor sense of direction showed lower levels of spatial knowledge when using a simple map relative to using no map or a complex map, suggesting that they did not attempt to use the complex map. For both groups of participants, referring to a simple map while learning a route decreased their awareness of their environment, as measured by response times on a memory test that included incidentally learned items.

## Significance

This research was prompted by the real-world challenges faced by international nuclear safeguards inspectors. These inspectors must develop accurate spatial knowledge about complex nuclear facilities, yet they have little control over the route that they take through the facility, they typically cannot use digital devices that could help them to track their location, and they may not have access to facility maps other than complex blueprints. In this study, we investigated three different types of paper-based facility maps, which reflect the types of maps that would typically be available to safeguards inspectors. We led participants on a route through a former nuclear facility and manipulated whether they studied a map before entering the facility or carried a map with them while learning the route. We tested various aspects of their spatial knowledge and their memory for items in the building. Our results suggest that simple maps are better for supporting spatial knowledge development in this context, but that the effects of map use vary considerably due to individual differences in sense of direction. We recommend that International Atomic Energy Agency (IAEA) inspectors self-assess their sense of direction, and that people with a low sense of direction might consider avoiding map exposure, as it does not seem to improve their spatial learning. In addition, since carrying a map during route-learning did not benefit spatial learning and had detrimental effects on participants’ attention to their environment, we recommend that inspectors avoid referring to maps during their inspection unless it is critical for their inspection duties.

International nuclear safeguards inspectors conducting facility inspections for the IAEA face interesting cognitive challenges when performing inspections in the field. They are led through a complex, often unfamiliar, nuclear facility by a host and have little to no control over their path through the facility due to safety, security, or operational constraints defined by their hosts. The main goals of their inspections are to detect the diversion of nuclear materials, the misuse of safeguarded facilities, and the development of undeclared nuclear facilities. Although inspectors typically have a pre-determined list of tasks that they need to accomplish during the inspection, such as collecting material measurements or verifying the design of the building, they are also expected to pay careful attention to their surroundings in order to identify potential anomalies that might warrant further verification activities under their international safeguards agreements or the Additional Protocol (International Atomic Energy Agency, 1997). As part of this overall awareness of their environment, inspectors are expected to monitor their route through the facility to ensure whether they have visited all of the rooms that they were supposed to visit, and detect whether certain areas of the building were avoided or hidden.

### Types of spatial knowledge

The main experimental question that this study sought to address was how to present building layout information to inspectors in a way that supports the development of their spatial knowledge of the environment but does not impede their ability to attend to other aspects of the environment. Inspectors must pay close attention to their environment as they move through a facility, both to ensure their safety as they move through a hazardous industrial environment, and to be on the lookout for any subtle cues that could indicate the diversion or misuse of nuclear materials or facilities. At the same time, they must build and maintain their spatial knowledge of the facility and their location within it. If the inspectors have more complete spatial knowledge of the facility, they will be better able to detect anomalies in the processes and operations of the facility, the layout of the building, or instances in which their guide leads them on circuitous routes, avoiding certain areas of the facility.

The question of what constitutes spatial knowledge is rather complex. It has been shown that there are at least three different levels of spatial knowledge: landmark, route, and survey knowledge (Siegel & White, 1975). Landmark knowledge refers to one’s memory for objects encountered in the environment (detached from the object’s location) and has recently been shown to develop in the absence of overt attention (van Asselen, Fritschy, & Postma, 2006). Route knowledge is defined as knowledge of an environment that is anchored by a series of actions taken at specific decision points. Route knowledge is more abstract that landmark knowledge and can contain the path between different landmarks, but is not thought to encompass aspects of the environment that were not encountered as part of a learned route nor to include metric knowledge about the distance between landmarks. Finally, survey knowledge is the most detailed representation of the relationships between all places in the environment. Survey knowledge represents the space from an allocentric point of view (i.e., relationships between places represented in terms of cardinal directions, angular degrees, or some other objective measurement) as opposed to an egocentric point of view as in route knowledge (i.e., turning left or right based on the individual’s location in the environment). Survey knowledge is marked by the ability to estimate straight-line directions and/or distances between landmarks, especially those that were never traversed between, and is believed to require effortful attention to generate.

Although Siegel and White (1975) originally suggested that these three levels of spatial knowledge develop sequentially, Montello (1998) proposed a competing framework suggesting simultaneous development of survey knowledge in parallel with the other levels of spatial knowledge. Indeed, Ishikawa and Montello (2006) demonstrated that large individual differences exist in the type of spatial knowledge that individuals learn about a novel environment after repeated exposures: some people developed survey knowledge almost immediately, some people never did, and still others showed a continuous progression in their knowledge. Similarly, Hölscher, Meilinger, Vrachliotis, Brösamle, and Knauff (2004) found that even people who were highly familiar with a particular building showed poor survey knowledge, even if they had excellent route knowledge. The evidence from these prior studies suggests that one model of spatial knowledge development may not fit all learners in all situations.

### Influence of learning conditions on spatial knowledge development

Some work exists in the cognitive science literature regarding what types of learning conditions best support the development of spatial knowledge, although none of it specifically addresses the constraints faced by IAEA inspectors. For example, it has been shown that being passively led along a route leads to worse spatial knowledge than if the individual has active control over their navigation (for review, see Chrastil & Warren, 2012). As such, IAEA inspectors already start at a disadvantage because they have no choice but to be passively led through the facility. Moreover, much of the cognitive science literature considers spatial learning and testing that takes place exclusively in a virtual environment (e.g., Carassa, Geminiani, Morganti, & Varotto, 2002; Chrastil & Warren, 2013, 2015), such as watching videos and being tested in a virtual environment (e.g., Meilinger, Knauff, & Bülthoff, 2008), learning maps of an environment without ever seeing it in person (e.g., Coluccia, 2008; Garden, Cornoldi, & Logie, 2002), or learning a route through a combination of virtual environments and still photographs (e.g., Gaunet, Vidal, Kemeny, & Berthoz, 2001). Since it is rarely possible to create virtual, video, or other digital representations of nuclear facilities, many of these findings from the cognitive science literature are not applicable to the IAEA. Similarly, there are tight restrictions on bringing electronic devices into nuclear facilities. While there have been several studies of digitally aided navigation and wayfinding (cf. Münzer, Zimmer, & Baus, 2012; Richter, Dara-Abrams, & Raubal, 2010; Schmid, Richter, & Peters, 2010; Schwering, Krukar, Li, Anacta, & Fuest, 2017), we cannot employ digital aids in this problem space. However, we can draw upon what other researchers have learned from comparisons of different methods of route learning.

There have been several studies that have compared the efficacy of learning a pre-determined route from a map versus other sources. In studies that have compared learning a route from a map versus learning through direct experience (typically by following an experimenter along a guided route), two studies have found approximately similar levels of spatial learning under both conditions (Ishikawa, Fujiwara, Imai, & Okabe, 2008; Richardson, Montello, & Hegarty, 1999). However, Ishikawa et al. (2008) found that the map group walked more slowly and made more errors than the direct experience group. This is especially relevant to IAEA safeguards inspectors because they have only a limited amount of time to complete their verification activities due to their global verification obligations as well as pressure from the nuclear facility operators to complete their activities quickly, since these activities usually cause a disruption to operational activities and, therefore, lost revenue.

Additionally, Richardson et al. (1999) noted alignment effects in the map condition (Levine, Marchon, & Hanley, 1984), in which pointing errors were higher when the participant was misaligned in space with the original orientation of their map, suggesting that people build an orientation-specific representation when learning from a map. Thorndyke and Hayes-Roth (1982) found better survey knowledge in map learners, but better procedural knowledge (i.e., routes between locations) in their direct experience group. However, their direct experience group worked in the building where the testing took place, so their effects could be due to prolonged exposure to the environment prior to the experiment.

Other studies have compared learning a route from a map versus learning from a GPS device. While the use of GPS is not an option for IAEA safeguards inspectors due to limited functionality of these devices indoors and restrictions on the use of electronic devices in nuclear facilities, GPS-aided spatial learning is similar to the guided navigation that safeguards inspectors experience while being led through a facility. Research in this area has shown that GPS tends to produce worse spatial learning outcomes, particularly on complex parts of the route (Ishikawa et al., 2008; Willis, Hölscher, Wilbertz, & Li, 2009). Willis et al. (2009) posited that the piecemeal way in which the map was displayed to participants in the GPS condition may have contributed to a more fragmented knowledge of the configural layout of the environment, relative to the map condition in which the entire environment was visible at once.

Li, Brown, Pinchin, and Blakey (2015) compared learning a route through a complex, indoor environment (a hospital) from a map versus a verbal description and found that participants learned the route equally well from both sources, although the map group was able to walk the route in reverse faster than the verbal description group.

Other studies have assessed the impact of different types of GPS-style wayfinding aids on spatial learning. For example, Löwen, Krukar, and Schwering (2019) compared map schematizations that emphasized different types of features along a route. The maps could emphasize local features (such as local landmarks), global features (structural features, such as city or area boundaries), or both. They found that accentuating local features improved participants’ route knowledge, but not their survey knowledge, while accentuating global features improved participants’ survey knowledge, but not their route knowledge. Similarly, Münzer et al. (2012) found tradeoffs between learning routes and learning configural information when participants used mobile navigation assistance systems that presented different types of information. When the presentation mode provided configural information, participants had better configural knowledge, but poorer wayfinding performance. When the presentation mode emphasized the route, participants had better wayfinding performance, but poorer configural knowledge. The researchers also found that individual differences in the participants’ sense of direction had a substantial impact on both types of learning.

Several studies have compared learning a route from a map versus pictures and/or videos of the route. In the same study just described, Li et al. (2015) found that people were better able to learn a route through a hospital from a video of the route than a map alone, as measured by their speed in traversing the route both forward and backward, although there were no differences in the number of errors made. Münzer and Stahl (2011) compared learning an indoor route from a map, egocentric photos of the route, or an egocentric video of the route. In terms of errors made, the map did not differ from the combination of the two egocentric conditions; however, the map condition did induce more hesitations than the video. Across these two studies, it seems that the ability to reproduce a specific route in an indoor environment is roughly equally supported by both maps and pictures/videos, with the caveat that the maps may induce higher cognitive load, as evidenced by taking longer to complete the route or showing more hesitations along the way. However, neither of these studies probed other aspects of spatial learning, like survey knowledge of the environment, so we cannot say how these two learning conditions extend beyond reproducing a specific route.

At least two studies have compared learning an indoor route from a map versus a virtual environment. Richardson et al. (1999) found the worst learning outcomes from their virtual environment compared to their map and direct experience conditions. On the other hand, Bliss, Tidwell, and Guest (1997) measured the ability of firefighters to reproduce a route through an unfamiliar building after learning from a map or from a virtual walkthrough of the route. They found equally good performance between the two conditions in terms of both time to complete the route and number of errors made. However, one caveat to note is that these studies are both at least 20 years old, and so it could be the case that newer, more immersive virtual reality environments may show different effects if compared directly to learning from maps.

To our knowledge, only a handful of previous studies directly compare the efficacy of different types of paper or non-interactive maps on wayfinding or spatial knowledge development. In one such study, Dillemuth (2005) tested whether participants were better able to learn an outdoor route from viewing a detailed aerial photograph versus a “generalized” map of a college campus (in which the buildings, grass, paths, etc., were filled in with a solid color). The maps were statically displayed on a handheld electronic device that did not include GPS to update position or orientation. They found that subjects performed better on their wayfinding measures when using the simple generalized map, particularly on time to route completion and number of stops made on the route. They also found that users did more zooming in and out when using the more detailed map, suggesting that more attention was required to use the detailed map. Interestingly, participants with higher sense of direction (as measured by the Santa Barbara Sense of Direction Scale, or SBSOD) performed much better with the simple than detailed map, contrary to what may be predicted.

In another study, Liben, Myers, and Kastens (2008) asked whether different types of map shapes (i.e., circular or square) and angle (i.e., drawn from directly overhead or at an oblique angle to display the buildings in slight relief) enabled college students to more accurately locate themselves during a route-learning task. They found no differences in accuracy based on map type, but they did observe response-time differences in which the square oblique map was the fastest and square flat maps were slowest. This was interpreted as showing that participants generated the strongest spatial knowledge when using the square oblique map. Additionally, they found that people were less likely to rotate the square map to match their orientation, which could have contributed to alignment effects in their results. Unfortunately, neither of these studies measured other types of spatial knowledge (i.e., landmark or survey knowledge), so we cannot say for sure whether either of these map types supported the development of spatial knowledge more generally, or whether they were simply better suited to the tasks assessed.

In a study of map schematization, Meilinger, Hölscher, Büchner, and Brösamle (2007) tested participants’ ability to localize themselves and complete wayfinding tasks inside of a complex building, using either a standard floor plan or highly schematized maps that provided only route information. For the self-localization tasks, the participants performed equally well with either type of map. For the wayfinding tasks, participants performed better when using a schematized map. While the highly schematized maps were useful for wayfinding, they explicitly excluded survey information. For the IAEA safeguards inspectors, developing survey knowledge is extremely important, since they must be able to understand how the rooms in a facility relate to one another and to the route on which they are guided by the facility operators. Therefore, although schematized maps may be useful for indoor navigation in other contexts, they would not support the cognitive needs of the safeguards inspectors.

### Current study

The current study is unique in that we are testing participants in a former nuclear hot-cell facility, which constitutes an extremely complex, indoor environment that was designed around safety and operational requirements rather than easy human navigation. In this way, we ensure that many of the physical and visual characteristics of the environment are well-matched to those that will be faced by safeguards inspectors in the field. This is also true of the experimental conditions that we tested. We tested three types of maps that are representative of the types of maps that safeguards that inspectors have access to when visiting a facility. We obtained actual facility maps from the group that manages the building in which our experiment took place. One map was a simple facility map, intended for space management purposes. The second map was a blueprint of the facility, which was highly complex and included a great deal of extraneous detail. The third map was a 3D representation of the simple facility map, created using the widely available software tool, (SketchUp [Computer Software], 2018) (www.sketchup.com). All three of the maps showed the overall layout of the building accurately, but all three also had minor inconsistencies between the map and the building, due either to changes that had taken place in the building over time (such as the removal of a door) or a lack of detail in the map (such as a temporary rolling partition not appearing on the map). These minor inconsistencies between a map and a facility are highly common in industrial facilities, where the building’s usage changes over time, but older maps are still in use. All maps were provided to participants on standard-sized paper because IAEA inspectors are typically unable to bring electronics into safeguarded facilities. As such, interactive or digital maps were not included as test conditions.

Our experimental conditions allowed us to ask several questions that are unique relative to the existing literature. First, we asked whether studying a map of the building prior to completing a guided route-learning task improves spatial learning. Next, we asked whether the ability to carry and refer to a map when learning a guided route provides an additional benefit to spatial knowledge, beyond the benefit of studying the map beforehand. Finally, we asked whether the level of detail in the map impacted spatial learning. We predicted that studying a map before the route-learning task would improve spatial knowledge, and that carrying the map during the route-learning task would provide an additional benefit. We also predicted that participants would receive a greater benefit from the simpler maps than from the complex blueprint, which was difficult to read and contained extraneous, distracting information.

We measured learning at every level of spatial knowledge (i.e., landmark, route, and survey), in addition to testing the participants’ awareness of details in their environment via a non-spatial memory test. We chose tasks that have been shown to reflect landmark knowledge (i.e., a landmark recognition memory task; Wenczel, Hepperle, & von Stülpnagel, 2017), route knowledge (i.e., drawing the guided route on an outline of the building; Labate, Pazzaglia, & Hegarty, 2014), and survey knowledge (i.e., a verbal pointing task, indicating angular direction between landmarks; Rand, Creem-Regehr, & Thompson, 2015). We also chose tasks that simultaneously tapped into multiple levels of spatial knowledge, including filling in a building outline with the name and location of learned landmarks and navigating a novel shortcut between two landmarks (Labate et al., 2014). The non-spatial memory task was designed to assess participants’ ability to maintain awareness of their environment by testing their recognition of incidental landmarks (i.e., van Asselen et al., 2006; Wenczel et al., 2017).

We also included a measure of individual differences in sense of direction, given the large differences in spatial knowledge acquisition found between people with good and poor senses of direction (Hegarty, Richardson, Montello, Lovelace, & Subbiah, 2002; Münzer et al., 2012; Wolbers & Hegarty, 2010). We administered the SBSOD (Hegarty et al., 2002), which is a 15-question scale that has been shown to be highly correlated with measures of spatial knowledge acquired from direct experiences in an environment. This allowed us to test whether the map study conditions had differential impacts on the development of spatial knowledge in individuals depending on their sense of direction. We predicted that participants with a poorer sense of direction would have lower levels of spatial knowledge, even with the aid of the maps.

## Method

### Participants

The participants were recruited from the employee population of Sandia National Laboratories (SNL), but people who were familiar with the building in which the study took place were excluded from participation. One hundred and twenty-three participants took part in the study; however, two of these were dropped for having excessively high errors on one critical experimental task (verbal pointing), and one participant was excluded due to building access issues during their data-collection session (i.e., access to a room on the route was blocked). After these participants were excluded, 120 participants were included in the final dataset (47 women). Women were roughly evenly distributed across the experimental conditions. There were 24 participants in each of the five map conditions, with either nine or ten female participants in each group. The mean age of the participants was 36.9 years (range: 18–69 years). None of the participants had worked as safeguards inspectors, but many came from technical backgrounds that are similar to that of the inspectors. They had a wide range of educational histories. The highest level of education reported was high school for four participants, some college for seven, an associate’s degree for seven, a bachelor’s degree for 24, some graduate school for three, a masters degree for 59, a JD for one, and a PhD for 14.

The experimental protocol was approved by the SNL Human Studies Board, and all participants gave informed consent before beginning the experiment. Because the building was a former nuclear facility, much care was taken to ensure that there were no ongoing safety concerns for participants or experimenters (i.e., risk of radiological exposure). The participants were compensated at their usual hourly pay rate for the time they spent participating in the experiment.

### Materials

#### Environment

**Fig. 1:**
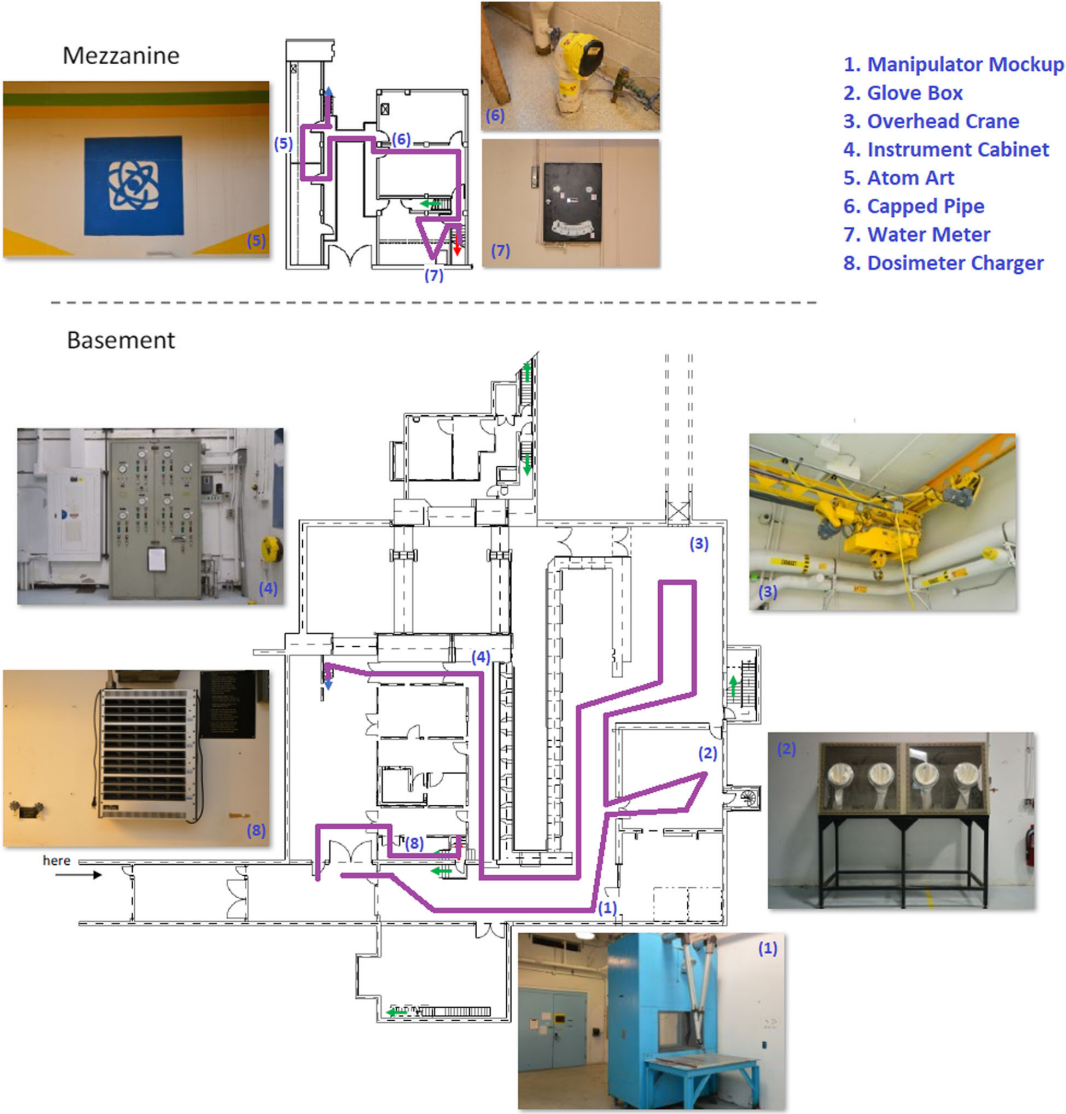
The simple study map overlaid with a line indicating the learned route and photos of the target landmarks

A former hot-cell facility on SNL’s campus served as the learning environment. Participants learned a route covering approximately 645 ft through the basement and mezzanine levels of the building. The route was a loop, so it started and ended at the same location. There were eight target landmarks along the route that participants were instructed to learn: five in the basement and three on the mezzanine level. None of the landmarks appeared on any of the maps, but rather were objects that were visible within the areas visited along the route. The landmarks were chosen to be distinctive and consisted of the following: a large blue manipulator, a set of glove boxes, an overhead crane, an instrument cabinet, an atom painted on the wall, a curved pipe coming out of the floor, a wall-mounted water meter, and a wall-mounted charger for dosimeters. Each of the target landmarks was given a two-word name (e.g., manipulator mockup, dosimeter charger) that the experimenter used to point out the landmarks during the navigation phase. Figure 1 shows the simple map of the building, overlaid with a depiction of the route and photographs of the eight landmarks.

Eight additional landmarks were selected to serve as incidentally learned items. These items were not explicitly pointed out to the participants. They were roughly equally spaced around the target landmarks, with five in the basement and three in the mezzanine level. The incidental landmarks were also distinctive in the environment and consisted of the following: a shelf of canisters, a different glove box, an electronic pressure meter on the hot cells, an elevated door without steps beneath it, a large indicator panel, a different overhead crane, a cubicle wall with a hole cut in it, and a series of tubes mounted on the wall). Finally, a set of eight distractor landmarks was chosen from parts of the building that were never visited by the participants. These landmarks maintained the industrial look and feel of the other landmarks (i.e., an emergency shower, a set of pipes with colored tops, an electrical box, a wall with a radiological caution sign, a piece of equipment, a stack of crates, a set of cabinets, and another tall cabinet of dials).^1^Photographs of all 24 landmarks were used in the landmark recognition task.

#### Maps

##### Study maps

Three different maps of the building were used for the study phase of the experiment: a simple map, a complex map, and a SketchUp map, which was a three-dimensional representation of the simple map. Each type of map will be described in turn below.

**Fig. 2:**
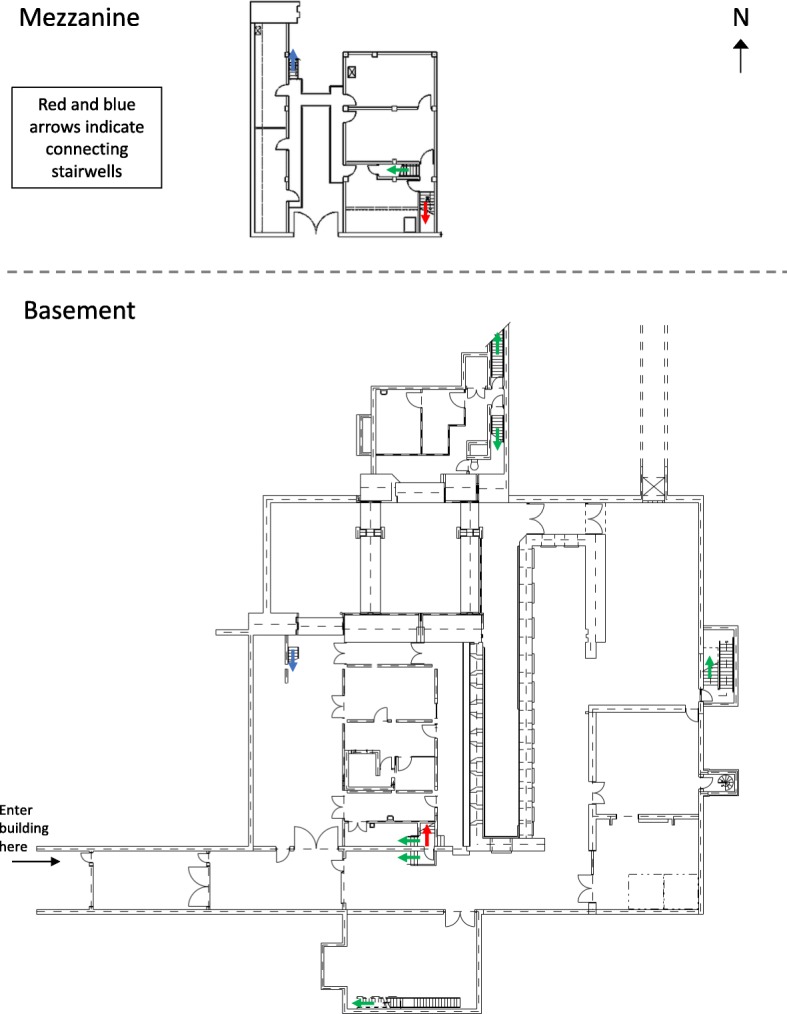
Study map for simple map carry and simple map study conditions, as seen by participants

The simple map is shown in Fig. 2. Participants in the “simple map study” and “simple map carry” conditions (described in more detail below) received the same map. The map was a simplified blueprint which showed walls, doorways, stairwells, and unique architectural features such as the location of the building’s hot cells. The basement and mezzanine maps were presented on the same page, in portrait layout, with the basement on the bottom of the page and the mezzanine level on the top part of the page. The building was oriented with north facing up, which was indicated with an arrow on the upper-right-hand side of the page. The size of the mezzanine was set to scale with the basement. Color-coded red and blue arrows indicated the stairwells that connected the basement and mezzanine levels to each other, while all other stairwells were marked with green arrows. There was also a marker indicating which entrance participants used to enter the building.

There were some inconsistencies between minor details in the map and the current state of the building due to changes in the building’s use that have taken place over time. While the relative size and layout of the rooms were accurate on the map, some elements of the building had changed since the creation of the map. The differences between the simple map and the building’s current layout were as follows:

- A stairway had been removed, but is still shown on the map
- Three doors are blocked and are no longer functional
- A rolling partition blocks the view of one area
- A door that is shown on the map has been removed and is now an open hallway
- A curtain blocks the view of a storage area, and on the map the curtain appears to be a wall
- A wall that is shown on the map has been removed
- A section of the catwalk in the mezzanine has been removed and the catwalk is approximately 10 ft shorter than what is depicted on the map
- Two cubicles have been constructed in one of the rooms, but are not shown on the map

**Fig. 3:**
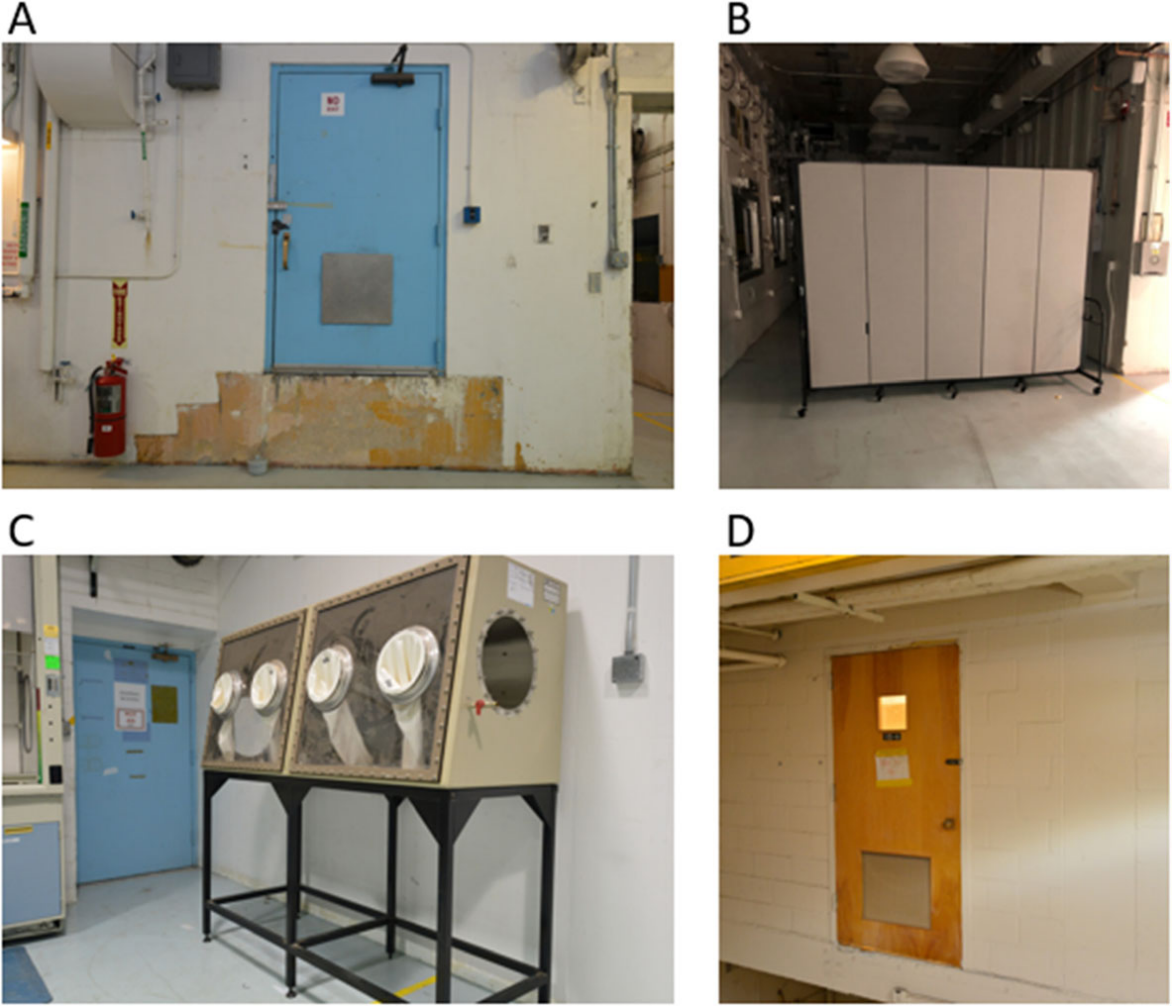
Photographs of some of the inconsistencies between the simple map and the building. Image **a** shows the stairs that have been removed and one of the non-functional doors. Image **b** shows the rolling partition, which is not depicted on the map. Image **c** shows another of the non-functional doors. Image **d** shows the third non-functional door and the area where the catwalk on the mezzanine has been removed. The sign on the door says, “Watch your step”

Photographs of some of these features, and how they appear in the building, are shown in Fig. 3. We did not anticipate that participants would notice the inconsistencies between the map and the building, with the exception of the wall that had been removed on the mezzanine level, since the route passed through the location where that wall used to be. The other inconsistencies were not directly relevant to the route. These inconsistencies between maps and as-built facilities are common in construction projects within any domain, and realistically represent the types of changes that may occur in a nuclear facility visited by IAEA safeguards inspectors.

**Fig. 4:**
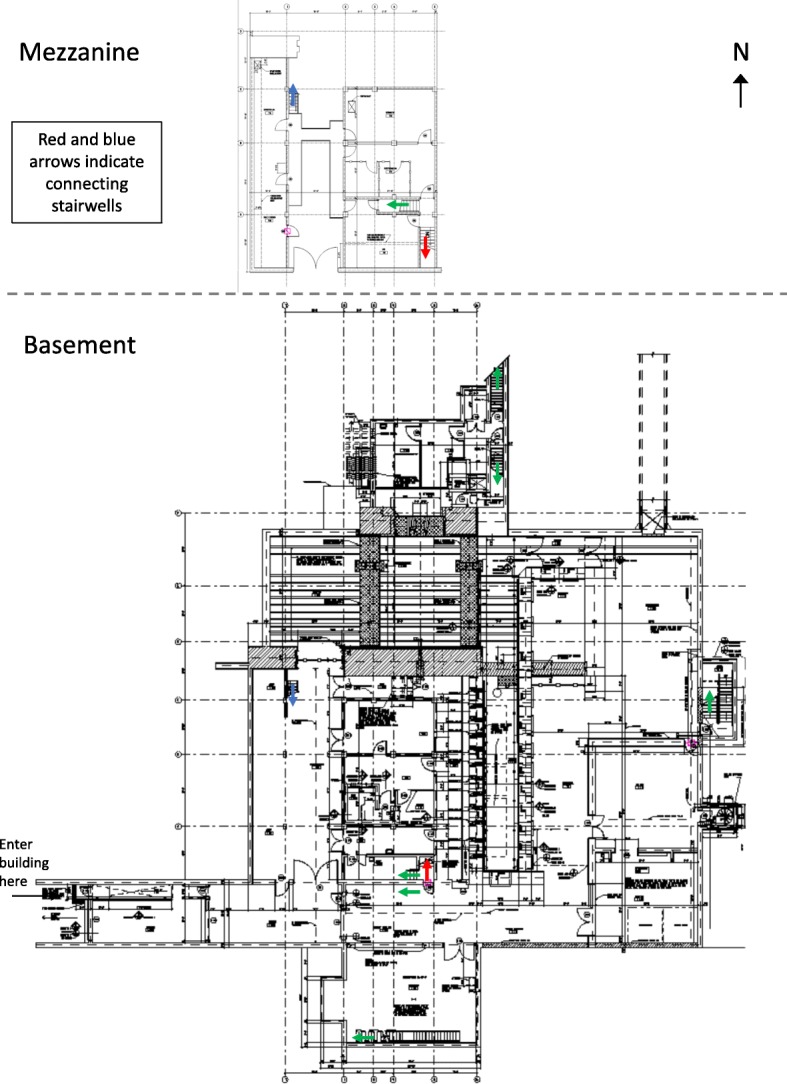
Study map for complex map carry condition

Participants in the “complex map carry condition” received a much more complex map of the facility, shown in Fig. 4. The complex map was oriented in the same way and contained the same experimenter-added markings as the simple map. This map contained many more details regarding the building, including additional markings indicating ductwork, measurements, and other extraneous information. The complex map was created more recently than the simple map, and thus there were fewer inconsistencies between the map and the building’s current status. For example, the non-functional doors are marked on the complex map, the length of the catwalk has been corrected, the cubicles have been added, the missing door and wall have been removed from the map, and the curtain and rolling partition are indicated with symbols that differ from those of the surrounding walls. The complex map provided the most details, so it gave the most complete representation of the building. This type of detailed blueprint is typical of the kinds of map that are most likely to be available to safeguards inspectors in the field.

**Fig. 5:**
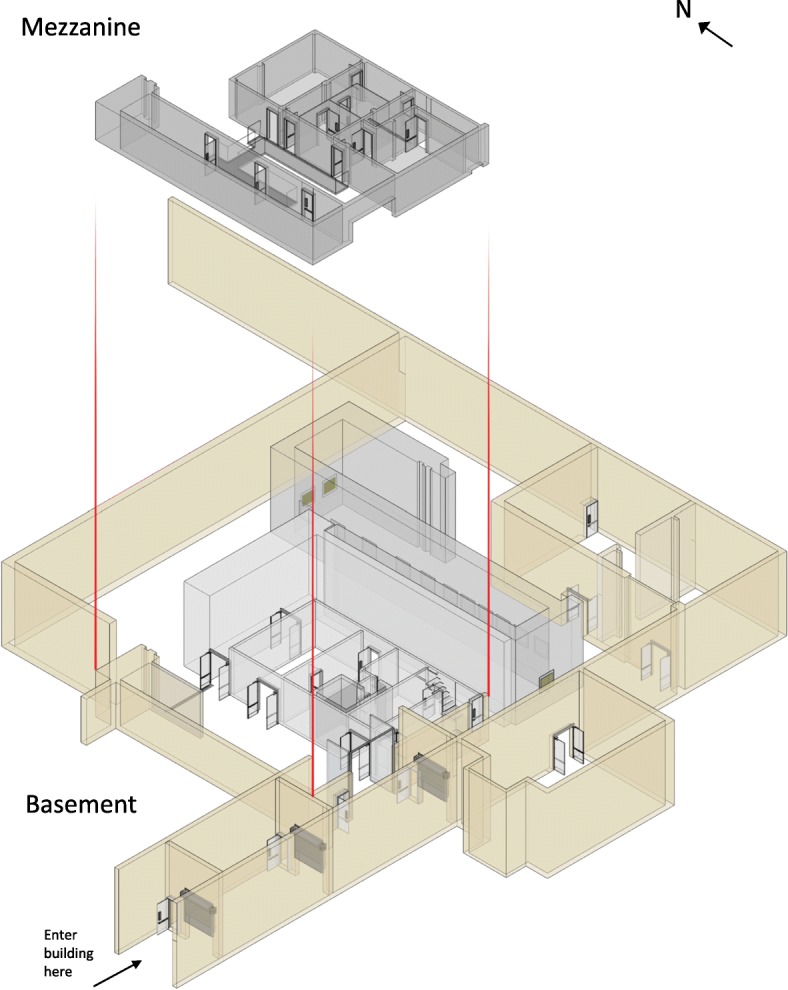
Study map for SketchUp carry condition

Participants in the “SketchUp map carry” condition received a 3D SketchUp version of the simple map, shown in Fig. 5. This map was created using the software tool (SketchUp [Computer Software], 2018) (www.sketchup.com). This map was rotated 45° (such that north faced the upper-left-hand corner of the paper) in order to allow for the visualization of perspective in the building and to ensure that the walls did not occlude one another. Various architectural features like walls, doorways, and stairwells were included in the map to create a simple 3D representation of the building. In this map, the non-functional doors were depicted as closed doors, while all other doors are open. The cubicles are shown, as is the correct length of the catwalk. The stairs, wall, and door that have been removed from the building are not shown. The curtain, rolling partition, and duct work are also not shown.

##### Test map

**Fig. 6:**
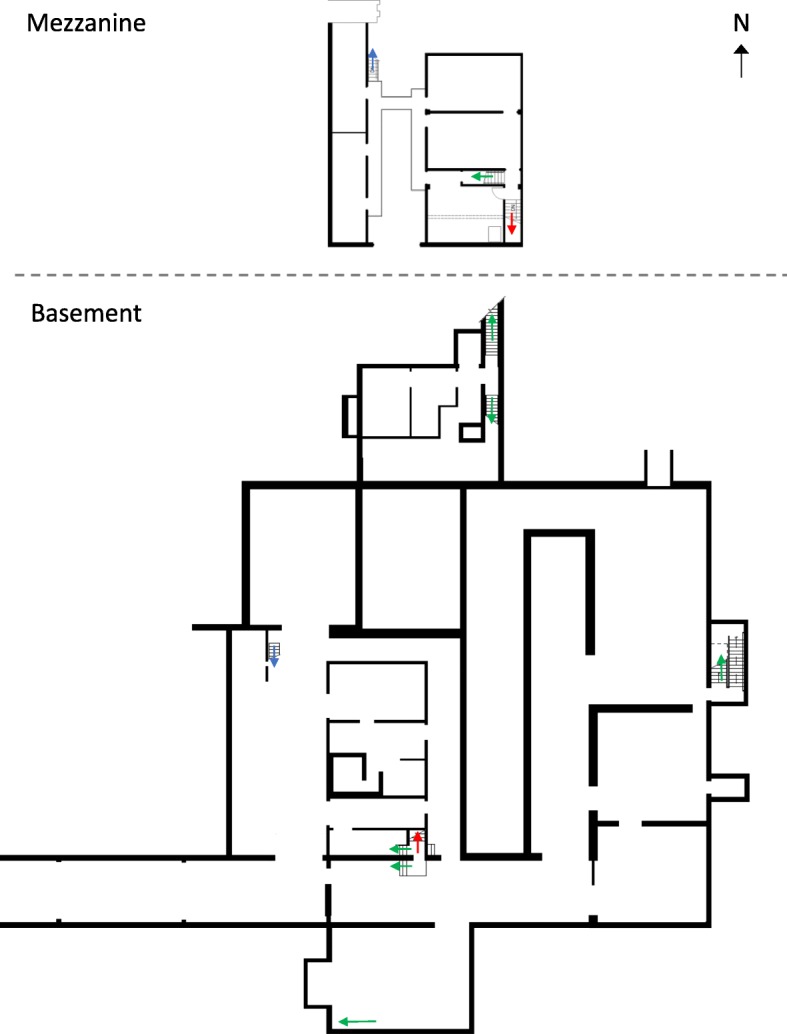
Test map for all conditions

At test, all participants were given a simplified test map, shown in Fig. 6, to ensure that all participants were equally unfamiliar with the appearance of the map presented at test. The test map was the same size and layout as the simple study map. However, all of the doors were removed, and the walls were filled in (such that unique architectural features were not visible on the test map). These changes were made to remove distinctive perceptual cues that might help participants on the map completion task, forcing them instead to rely on their knowledge of building layout.

##### Control materials

Participants in the “no-map” control condition received a two-page hand-out entitled “A day in the life of a safeguards inspector” (IAEA Bulletin, June 2016) to read during the study phase. This handout was chosen because the topic area was relevant to the experiment’s purposes, and also because it contained a small diagram in an infographic depicting a fictional route that an inspector may walk through a facility. In this way, control participants would not be able to guess that they were in the control condition.

#### Self-report questionnaires

##### Santa Barbara Sense of Direction Scale

The Santa Barbara Sense of Direction Scale (SBSOD; Hegarty et al., 2002) was administered to participants via E-Prime. The scale consists of 15 statements to which participants rate their agreement on a 7-point Likert scale (1 = Strongly Agree, 7 = Strongly Disagree). Seven of the statements are positive in valence (e.g., “I am very good at judging distances” and “I am very good at giving direction”), and eight of the statements are negative in valence (e.g., “I very easily get lost in a new city” and “I have trouble understanding directions”). The positive-valence questions are reverse-scored, and then all scores are summed to produce the final SBSOD score. A higher score indicates a better sense of direction.

##### Demographics and other self-report items

In addition to the SBSOD, participants were asked to provide basic demographic information (age, sex, and highest level of education). They were also asked to rate their familiarity with the testing environment prior to the experiment (to ensure that our exclusionary criteria were not violated) and their level of familiarity with reading/using blueprints and other technical design drawings of buildings. Participants answered these questions by providing a rating from 1 to 5, with 1 indicating “Not at all familiar” and 5 indicating “Extremely familiar.” Finally, participants were asked to rate how often they referred to the map (if they were in one of the map carry conditions) on a scale of 1–5, with 1 meaning “Never” and 5 meaning “Very frequently.”

### Procedure

#### Learning phase

Participants were tested individually. Because of access restrictions for the building where the experiment took place, experimenters met the participant at a sign-in kiosk in a nearby building, then escorted the participant to a conference room on the first floor of the test building. The basement and mezzanine levels of the building were both below the first floor, so participants did not see any part of the experimental route prior to beginning the experiment. After completing informed consent forms, the experimenter read a small blurb to the participant regarding international nuclear safeguards, in which participants were told that the purpose of the study was to understand how people learn the layout of a novel environment after different types of learning. Next, participants in the map conditions received their map of the building and were given 5 min to study the map. Participants were told that their job was to learn the layout of the two floors, with the caveat that the building may not match the map exactly. They were also informed that they were going to be led on a route through the building, along which they would learn landmarks, and at the end they would be tested on the landmarks, the building layout, and any differences they noticed between the map and the building. At this time, participants were also told whether or not they would be able to carry the map with them during the experiment. The control participants also received 5 min to study their handout and were given the same instructions regarding the upcoming task before their study period began. Twenty-four participants were assigned to each study condition.

After the 5-min study period, participants were led out of the building, down a set of stairs, and into the basement to the starting point of the route. Participants in the simple map carry, complex map carry, and SketchUp map carry conditions carried their maps with them during the route-learning phase. Participants in the no-map and simple map study conditions did not have a map to carry with them during this phase.

Participants began the route-learning phase by standing at the starting point of the route, which was indicated by a marking on the floor. Half of the participants traversed the route in one direction (roughly clockwise, beginning by turning to the north), and half in the other (roughly counterclockwise, beginning by turning to the east). Participants were asked to walk at the same pace as the experimenter and were told that they would not be able to ask questions during the route-learning phase. They were asked to use caution when going up and down the stairs and to observe all safety notices within the building. The experimenter walked slightly ahead of the participants to lead the way along the route. The experimenter paused at each landmark, pointed to it, and told participants its name. Then the experimenter paused there for about 3 s before continuing along the route. The route phase took approximately 3 to 5 min to complete.

#### Test phase

The test phase began immediately upon completion of the learning phase. All participants completed the tasks in the same order. If participants were in one of the map carry conditions, their map was taken away before they began the test phase. They were not allowed to refer to their map during any of the tests.

##### Verbal pointing task

**Fig. 7:**
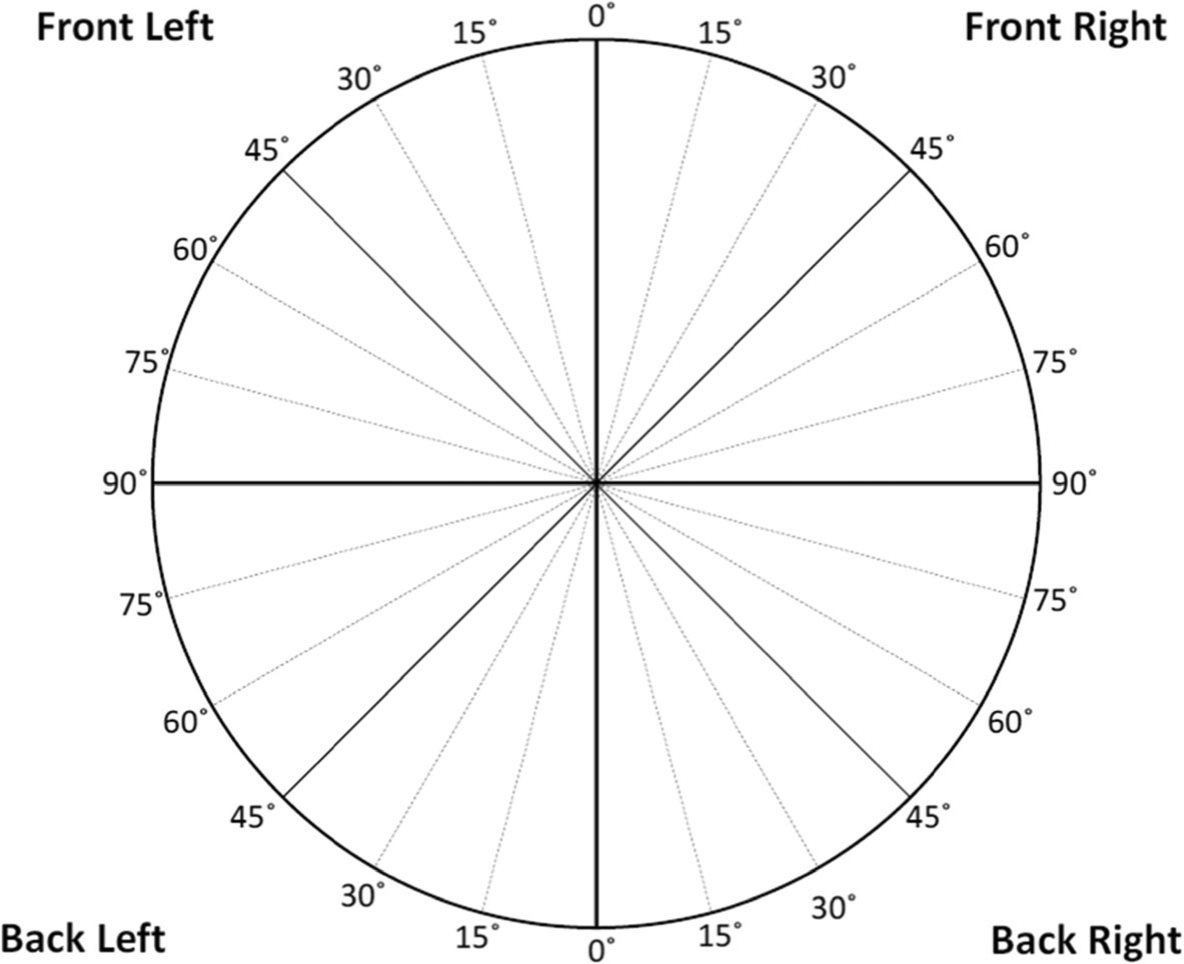
The response aid used for the pointing task

In the verbal pointing task, participants were positioned at the route starting/ending location and rotated 45° so that they faced directly northeast, halfway between the direction of the two routes’ starting directions. This orientation was marked on the floor to ensure that it was the same for all participants, and it was chosen to ensure that all participants were turned in a direction that was equally different from the direction they walked when beginning the route (as directional estimates have been shown to be most accurate when aligned with a participant’s starting direction). At the beginning of the task, participants were given a page with a circle divided into quadrants. The quadrants were labeled (e.g., “Front Right”) and the circle was marked in 15° increments, as shown in Fig. 7. They were asked to imagine that they were standing at the center of that circle, with the 0° line pointing straight ahead of them. The experimenter read the names of the eight landmarks aloud in a fixed random order. The participants used the circle as an aid to indicate the angle from their current orientation to the landmark. They were asked to imagine pointing to the landmark from where they stood, ignoring any intervening walls and floors, and then to indicate which direction they would be pointing. They reported their answers by first picking which quadrant of the circle they would be pointing towards, then estimating the angle in degrees within that quadrant. For example, a participant might say “Front Right quadrant, 22°.” The experimenter recorded the verbal responses on paper. If participants said that they did not remember the landmark, they were asked to make their best guess.

##### Shortcut task

In the shortcut task, participants were asked to walk the shortest distance between three pairs of landmarks. The landmark pairs were chosen such that they were not adjacent to each other on the route, and as such, there was a shorter path between them than the learned route through the building. The task started with the experimenter leading the participant to the first landmark and then naming their target landmark. Participants were instructed to walk to the target landmark using the shortest possible path. They were told that they could go through doors and rooms that were not entered during the route-learning task if they felt that would be a shorter path. For the first shortcut, they were asked to find the shortest path between the manipulator mockup and the dosimeter charger, which were the first and last landmarks they had encountered on their routes. Next, they were asked to find the shortest path between the dosimeter charger and the instrument cabinet. Finally, they were asked to find the shortest path between the instrument cabinet and the water meter. The first two pairs of landmarks were in the basement, and the last had one landmark in the basement and one landmark in the mezzanine.

The only feedback given to participants was to correct them if they started to cross one of four designated areas in the building beyond which there was no viable way to get to their target landmark. The experimenter would not describe the landmarks if the participant had forgotten them or give any hints about how to get there. Based on pilot testing, a maximum time of 3 min was allotted for the completion of each shortcut. Experimenters walked behind the participants and recorded the path walked through the building, the time elapsed before the participant started walking, the number of hesitations (pauses for longer than ~ 2 s after starting), the number of experimenter-corrected errors, and the total time (in seconds) that it took participants to reach the target landmark. If a participant had not found the target landmark after 3 min, the experimenter escorted them to it prior to beginning the next shortcut task.

After the shortcut task, participants returned to the conference room on the first floor of the building to complete the remaining tasks. There was a staircase near the final target landmark of the shortcut task that led directly to the conference room, so the participants did not have any additional exposure to the test environment or the learned route when leaving the area.

##### Map completion task

In the map completion task, participants received a simplified map of the building (see “Test map” description above) and were instructed to fill in: the starting/ending location of the learned route, the path of the route through the building, the names and locations of each of the landmarks, and any differences they noticed between the map and the building. Participants were allowed up to 5 min to complete this task.

##### Landmark recognition task

In the landmark recognition task, participants viewed pictures of objects in the building and indicated via button-press whether they had seen the object in the building or not. The test consisted of photos of the eight target landmarks, the eight incidental landmarks (which they walked by on the route but were not pointed out to them), and eight distractors (eight photos of objects from other parts of the building that the participants had never entered). The photos were presented to participants via E-Prime experimental software, and responses were made by pressing the arrow keys on the keyboard to indicate whether that had seen or not seen the landmark.

##### SBSOD

The SBSOD was administered electronically using E-Prime. Participants read each of the 15 statements and clicked on the Likert response that corresponded to their level of agreement with the statement.

##### Demographics and self-report questionnaire

For the final task, participants completed the demographics and self-report questionnaire, as described in the Materials section.

### Analysis

#### Verbal pointing task

Data from the verbal pointing task was scored as follows, following Rand et al. (2015). First, all values were converted to an angle from 0 to 360 (with 0 being straight ahead and increasing in degrees going clockwise). The difference between this value and the actual angle to the landmark was determined, and the absolute value of the non-reflex angle (the angle < 180°) of this difference was used as a measure of unsigned pointing error. Two participants were excluded because their average error exceeded 90°, again in line with Rand et al. (2015).

#### Shortcut task

The data from the shortcut task was measured in the same way as Labate et al. (2014) First, the length of the actual shortcut taken by each individual was determined (measured in centimeters on a scaled map of the building), and then the difference between the shortest possible shortcut and the actual shortcut was calculated. This value served as their shortcut distance error.

#### Landmark recognition task

For the landmark recognition task, two measures of interest were calculated. First, response times (RTs) were calculated for *correct responses* only. Second, the target discriminability measure *d’* was calculated separately for target and incidental landmarks (comparing both to the false-alarm rate for distractors). *d’* is more robust measure than simple accuracy, as it takes into account both the hit rate and false-alarm rate, and indicates a person’s ability to differentiate between items that they saw on the route and those that they did not. Since *d’* scores are indeterminate if a person has a perfect hit or false-alarm rate, we adjusted perfect scores to enable calculation of *d’*. If a person did not have any false alarms, they were given a score of 1/16, or .0625 (i.e., as if they had .5 of a false alarm). Similarly, if a person had a perfect hit rate, they were given a score of .9375 (i.e., as if they had .5 of a hit).

#### Map completion task

Two scores were calculated for the map completion task: a route score and a landmark score. First, for the route score, participants were binned into groups depending on whether they drew the route with few to no errors (less than three major errors, such as missing a room or a turn), with a moderate number of errors (approximately three to five major errors), or with a large number of errors (more than five major errors). The binning was completed by two experimenters who were blind to the map condition of the participant. The experimenters binned all of the participants, then discussed any instances in which they made different decisions for one participant. The experimenters than discussed the errors made by that participant until they reached an agreement about the proper binning. Participants received a score of 1 for a route with few to no errors, a score of .5 for a moderate number of errors, or a score of 0 if they had a large number of errors. Secondly, for the landmark score, participants could receive a total of 16 points: 1 point for correctly writing down the landmark’s name, and 1 point for drawing the landmark in the correct room. Half points were given if the name was partially correct (i.e., *mockup arms* for *manipulator mockup*), or if the landmark was erroneously placed in an adjacent room or in the wrong general part of the room (for larger rooms). The percentage of points received out of 16 was submitted for analysis.

#### Statistical analyses

The SBSOD scores were used to do a median split for the participants in each experimental group. Data from the pointing task, shortcut task, landmark recognition task (*d’* scores), and map completion task was analyzed using between-subjects Analysis of Variance (ANOVA) analyses, with five levels for the map condition factor (no map, simple map study, simple map carry, complex map carry, and SketchUp map carry) and two levels for the SBSOD factor (high or low).

## Results

### Descriptive statistics

Because previous work has shown significant effects of SBSOD in spatial knowledge tasks, we started by assessing whether our different map conditions were balanced with respect to SBSOD. The overall mean SBSOD score in our dataset was 70.03 (min = 31, max = 101, *SD* = 16.94). However, SBSOD did not differ across our experimental conditions (*F* (4,115) = 0.40, *p* = .81), the means of which are listed in Table 1. Due to our interest in understanding how an individual’s sense of direction interacts with their given map to influence their spatial learning, we created sub-groups within each condition based on a median split of all SBSOD scores (median = 72) to divide subjects into either high- or low-SBSOD categories.

**Table 1:** Mean Santa Barbara Sense of Direction (SBSOD) scores for each experimental condition

Condition	Mean SBSOD	Number of subjects below median split	Number of subjects above median split
No map	71.25	12	12
Simple map Study	66.58	14	10
Simple map Carry	70.50	12	12
Complex map Carry	72.38	8	16
SketchUp carry	69.45	13	11

We also analyzed the participants’ self-reported use of the maps in the three map carry conditions. These results are shown in Table 2. In general, participants reported that they referred to the maps at least occasionally during the route-learning task. Only five participants reported that they never referred to the map.

**Table 2:** Self-reported frequency of map usage during the route-learning task

	Simple map		Complex map		SketchUp map	
Response	High SBSOD	Low SBSOD	High SBSOD	Low SBSOD	High SBSOD	Low SBSOD
Never	0	0	1	2	0	2
Rarely	1	3	2	1	1	2
Occasionally	3	2	3	0	4	4
Frequently	4	4	7	3	2	2
Very Frequently	4	3	3	2	4	3

*SBSOD* Mean Santa Barbara Sense of Direction

### Pointing task

Mean pointing-error values (as well as mean scores for all of the other tasks) for each map study condition are listed in Table 3, with the values for the high- and low-SBSOD groups shown in Table 4. The ANOVA showed no main effect of map condition (*F* (4,110) = 0.91, *p* = .46), but a main effect of sense of direction (*F* (1,110) = 7.75, *p* < .01), and a significant interaction between the two (*F* (4,110) = 6.72, *p* < .01).

**Table 3:** Mean values and standard deviations (SDs) for each measure by map study condition

Measure	Condition	Mean	SD
Pointing task error	No map	38.4	37.4
	Simple map study	33.4	34.4
	Simple map carry	31.0	27.5
	Complex map carry	36.0	31.9
	SketchUp carry	33.9	32.6
Shortcut task distance error	No map	4.45	2.99
	Simple map study	5.49	4.35
	Simple map carry	4.71	4.85
	Complex map carry	5.14	3.76
	SketchUp carry	5.42	4.67
Landmark recognition task target Landmark *d*’	No map	2.49	0.52
	Simple map study	2.31	0.64
	Simple map carry	2.42	0.57
	Complex map carry	2.53	0.46
	SketchUp carry	2.51	0.40
Landmark recognition task incidental Landmark *d*’	No map	1.06	0.75
	Simple map study	0.91	0.64
	Simple map carry	0.85	0.69
	Complex map carry	0.91	0.45
	SketchUp carry	0.97	0.60
Map completion task route score	No map	0.42	0.43
	Simple map study	0.46	0.44
	Simple map carry	0.60	0.39
	Complex map carry	0.48	0.43
	SketchUp carry	0.48	0.48
Map completion task landmark score	No map	0.73	0.22
	Simple map study	0.75	0.24
	Simple map carry	0.75	0.23
	Complex map carry	0.72	0.20
	SketchUp carry	0.67	0.25

**Table 4:** Mean values and standard deviations (SDs) for each measure, split by map condition and sense of direction

Measure	Sense of direction	Condition	Mean	SD
Pointing task error	Low SBSOD	No map	33.57	31.07
		Simple map study	41.15	39.70
		Simple map carry	38.22	30.67
		Complex map carry	29.73	23.59
		SketchUp carry	43.69	37.68
	High SBSOD	No map	43.21	42.44
		Simple map study	22.68	21.40
		Simple map carry	23.83	21.75
		Complex map carry	39.14	35.05
		SketchUp carry	22.26	20.02
Shortcut task distance error	Low SBSOD	No map	3.70	2.42
		Simple map study	6.67	4.71
		Simple map carry	6.30	5.22
		Complex map carry	3.10	1.50
		SketchUp carry	6.54	5.45
	High SBSOD	No map	5.19	3.41
		Simple map study	3.83	3.32
		Simple map carry	3.13	4.07
		Complex map carry	6.16	4.15
		SketchUp carry	4.09	3.30
Landmark recognition task Target landmark *d’*	Low SBSOD	No map	2.66	0.32
		Simple map study	2.12	0.65
		Simple map carry	2.28	0.56
		Complex map carry	2.46	0.60
		SketchUp carry	2.54	0.39
	High SBSOD	No map	2.31	0.64
		Simple map study	2.58	0.53
		Simple map carry	2.54	0.58
		Complex map carry	2.56	0.39
		SketchUp carry	2.48	0.43
Landmark recognition task Incidental landmark *d*’	Low SBSOD	No map	1.14	0.78
		Simple map study	0.87	0.71
		Simple map carry	0.64	0.76
		Complex map carry	0.93	0.59
		SketchUp carry	0.96	0.59
	High SBSOD	No map	0.98	0.74
		Simple map study	0.96	0.56
		Simple map carry	1.03	0.59
		Complex map carry	0.90	0.38
		SketchUp carry	0.99	0.65
Map completion task route score	Low SBSOD	No map	0.46	0.45
		Simple map study	0.25	0.38
		Simple map carry	0.50	0.37
		Complex map carry	0.75	0.38
		SketchUp carry	0.23	0.39
	High SBSOD	No map	0.38	0.43
		Simple map study	0.75	0.35
		Simple map carry	0.71	0.40
		Complex map carry	0.34	0.40
		SketchUp carry	0.77	0.41
Map completion task landmark score	Low SBSOD	No map	0.73	0.19
		Simple map study	0.63	0.23
		Simple map carry	0.64	0.28
		Complex map carry	0.83	0.20
		SketchUp carry	0.54	0.25
	High SBSOD	No map	0.72	0.27
		Simple map study	0.93	0.12
		Simple map carry	0.85	0.12
		Complex map carry	0.67	0.17
		SketchUp carry	0.82	0.15

*SBSOD* Mean Santa Barbara Sense of Direction

#### High-SBSOD group

**Fig. 8:**
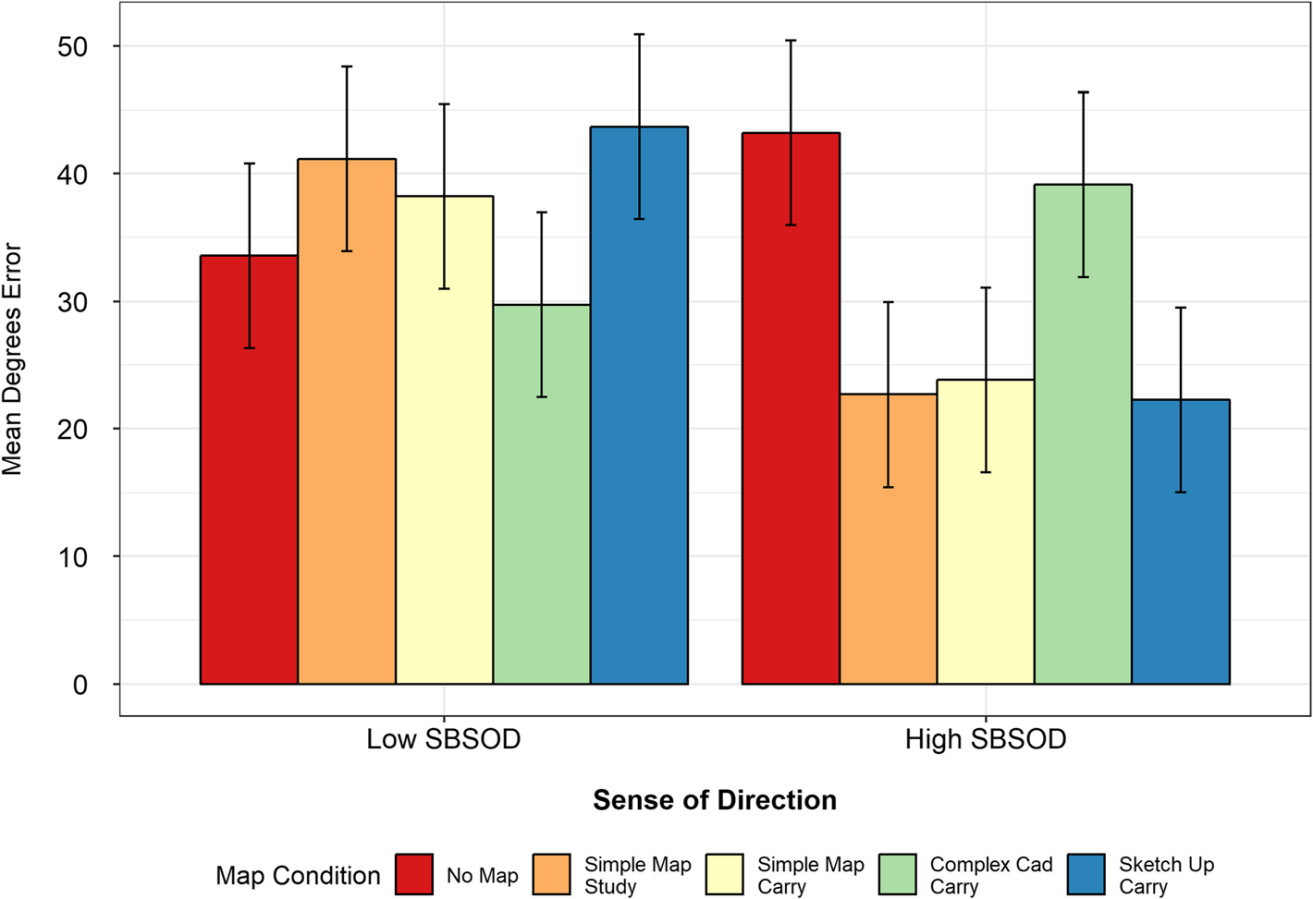
Mean pointing error across map conditions by median split of Mean Santa Barbara Sense of Direction (SBSOD). Error bars in this and all subsequent figures are 95% confidence intervals

Follow-up *t* tests found that the no-map and complex map carry conditions elicited the largest pointing errors for the high-SBSOD group (see Fig. 8). The mean pointing error was significantly larger for the no-map condition than for the simple map study (*t* (17.69) = 4.35, *p* < .01), simple map carry (*t* (16.66) = 4.23, *p* < .01) or SketchUp carry (*t* (18.62) = 4.33, *p* < .01) conditions. Similarly, the mean pointing error was significantly higher for the complex map carry condition than for the simple map study (*t* (22.55) = 3.37, *p* < .01), simple map carry (*t* (21.65) = 3.22, *p* < .01), or SketchUp carry (*t* (23.55) = 3.37, *p* < .01) conditions. The similar results for the no-map and complex map conditions suggest that studying and carrying the complex map provided no additional benefit relative to having no map at all. While the complex map did not benefit the participants, studying and/or carrying the simple and SketchUp maps improved performance on the pointing task.

#### Low-SBSOD group

The low-SBSOD group had a very different pattern of performance than the high-SBSOD group. Their mean pointing error was significantly *better* in the complex map carry condition than in the simple map study condition (*t*(19.77) = 2.16, *p* = .04). There were no significant differences when comparing the other conditions. However, their performance was numerically best for the conditions in which the high-SBSOD participants performed the worst: the no-map and complex map carry conditions. Their lower pointing errors for the complex map carry condition approached the threshold for statistical significance when compared to the simple map carry (*t*(16.90) = 2.10, *p* = .051) and SketchUp map carry (*t*(16.33) = 2.01, *p* = .06) conditions.

When comparing the groups to one another, there was not a significant difference between the low- and high-SBSOD groups for the no-map (*t*(21.82) = 1.61, *p* = .12) or complex map conditions (*t*(22) = 1.83, *p* = 0.08). However, the high-SBSOD group performed significantly better than the low-SBSOD group for the simple map study, simple map carry, and SketchUp map conditions (all *t*s > 3.13, all *p*s < .01). These results indicate that the groups had similar levels of performance for the no-map and complex map conditions, and while the participants in the high-SBSOD group benefited from studying or carrying the simple or SketchUp maps, the participants in the low-SBSOD group did not.

### Shortcut task

Although multiple measures were collected for the shortcut task (i.e., shortcut distance error, time to complete shortcut, number of experimenter-corrected errors), most of these measures patterned together. Thus, for brevity and consistency with the original study on which the shortcut task was based (Labate et al., 2014), we will only report the shortcut distance error.

**Fig. 9:**
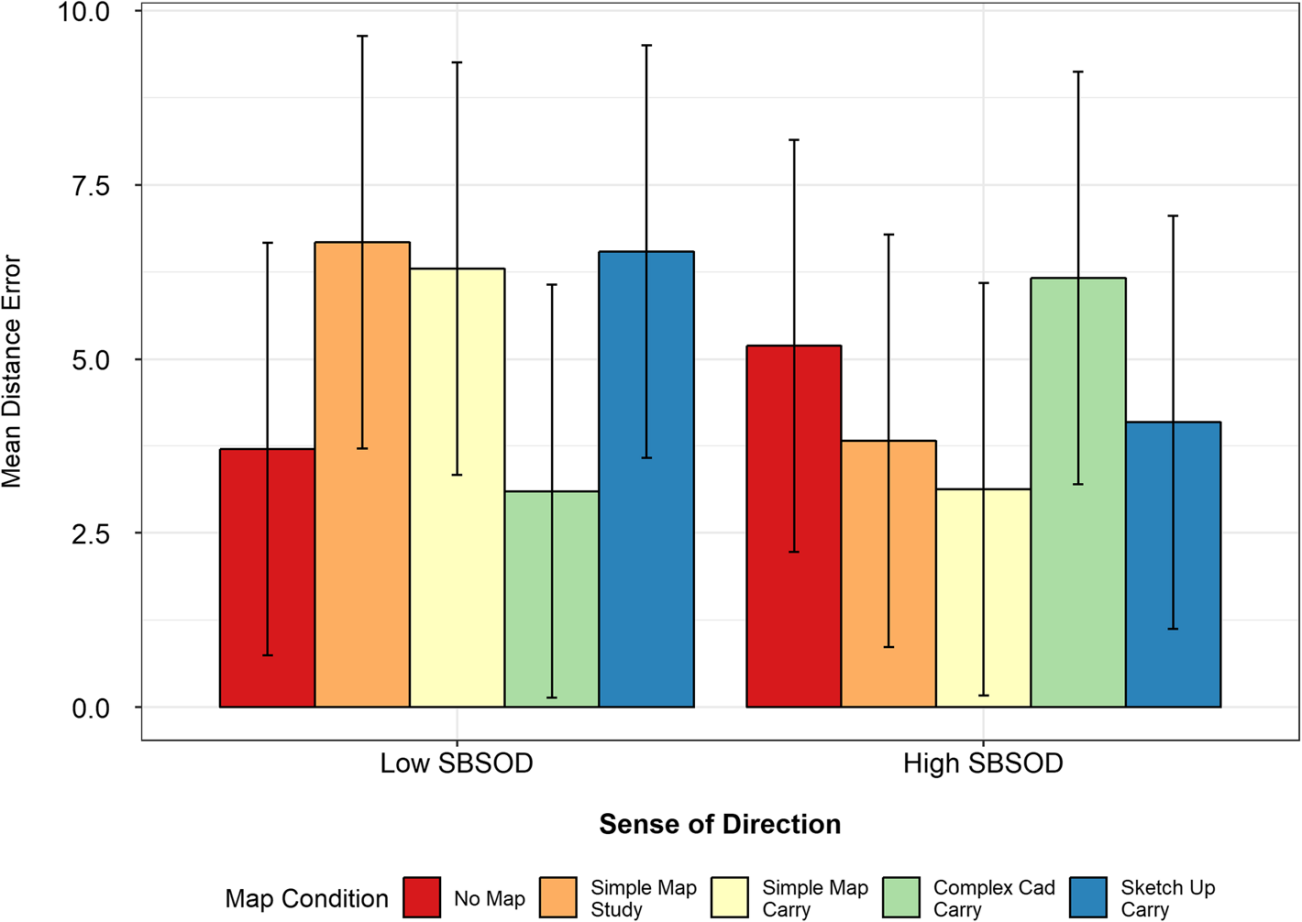
Shortcut distance error shown as a function of map condition for high- versus low-Mean Santa Barbara Sense of Direction (SBSOD) groups

The analysis of the shortcut distance error did not show an effect of map condition (*F*(4,110) = 0.30, *p* = .88) or sense of direction (*F*(1,110) = 1.30, *p* = .26), but did show a significant interaction between the two (*F*(4,110) = 2.82, *p* = .03). The results are shown in Fig. 9.

#### High-SBSOD group

For the high-SBSOD participants, there were no significant differences across the map conditions due to the high variability on this measure. Numerically, the participants had larger errors for the no-map and complex map carry conditions.

#### Low-SBSOD group

As with the pointing task, the no-map and complex map carry conditions produced the best scores for the low-SBSOD group, despite producing the worst scores for the high-SBSOD group. Low-SBSOD participants in the complex map carry condition had better performance (lower mean errors) than participants in the simple map study (*t*(17.04) = 2.62, *p* = .02) and the SketchUp carry conditions (*t*(14.76) = 2.15, *p* < .05). The difference also approached the threshold for significance when comparing the complex map to the simple map carry (*t*(13.57) = 2.00, *p* = .07) condition, as well as when comparing the no-map condition to the simple map study condition (*t*(20.01) = 2.06, *p* = .052).

### Landmark recognition task

#### *d’* Scores

Target discriminability, or *d’*, scores were calculated separately for target items, which were the landmarks pointed out to participants along the route and on which participants were tested in the pointing task, and incidental items, which were visible along the route but were not explicitly pointed out to participants. We will consider them separately to understand how well the different map conditions impact one’s ability to overtly remember critical parts of the building, versus incidentally encoded items that reflect participants’ ability to maintain awareness of their environment during the route-learning task.

The mean *d’* scores for the target and incidental items are shown in Table 3. For the target items, we did not observe main effects of map condition (*F*(4,109) = 0.72, *p* = .58) or sense of direction (*F*(1,109) = 0.66, *p* = .42), nor a significant interaction between the two (*F*(4,109) = 2.14, *p* = .08). Similarly, for the incidental items, there was no main effect of map condition (*F*(4,109) = 0.39, *p* = .82), no main effect of sense of direction (*F*(1,109) = 0.27, *p* = .60), and no interaction (*F*(4,109) = 0.61, *p* = .66).

#### Response times

**Fig. 10:**
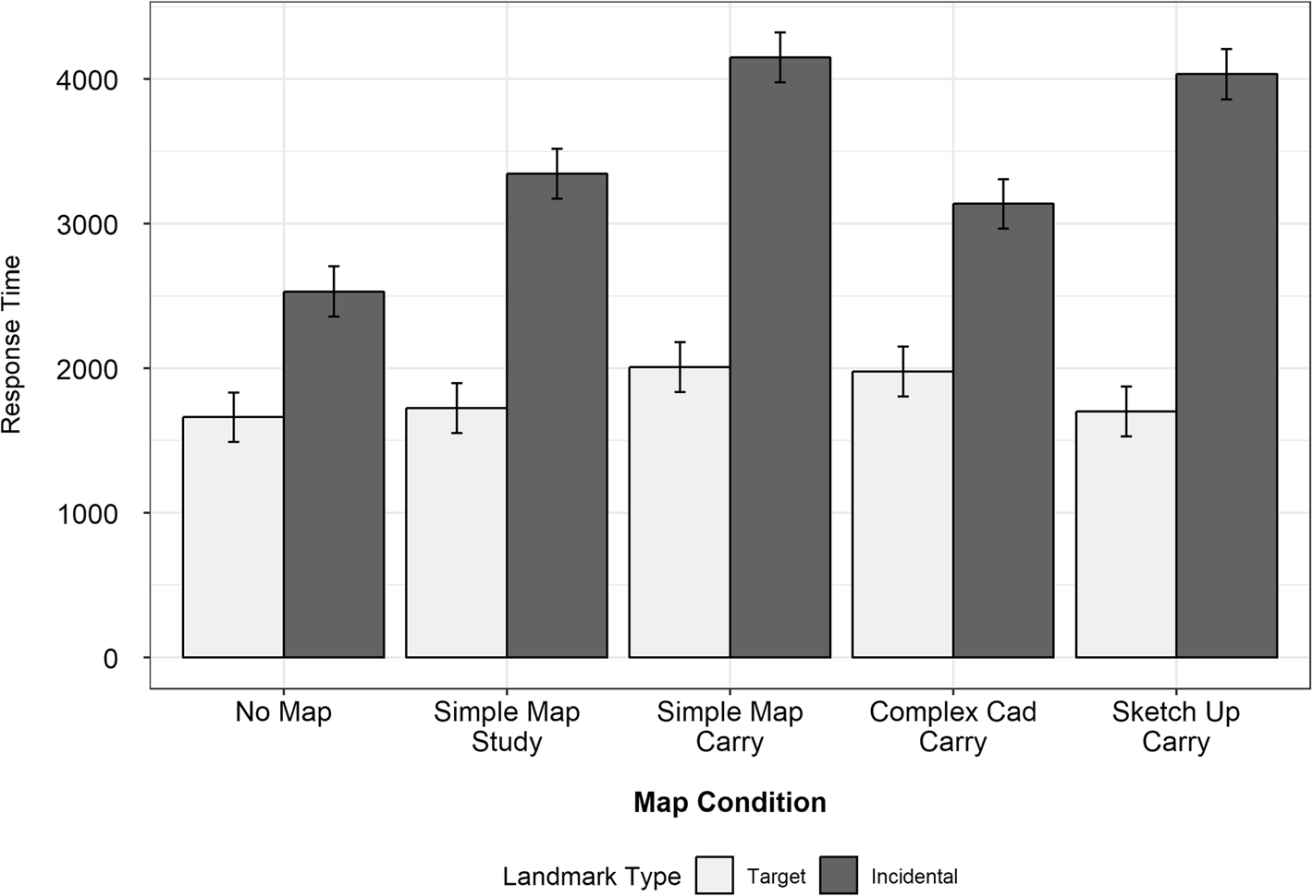
Response times for correct responses to landmark recognition test trials, split by landmark type and map condition

The RTs were calculated for the target and incidental items, including correct responses only. The mean RTs are shown in Fig. 10 and listed in Table 5. For the target items, there was no effect of map condition, sense of direction, or interaction between map condition and sense of direction for the target landmark RTs (all *F*s < 1.83, all *p*s > .13). For the incidental items, there was a main effect of map condition (*F*(4,109) = 2.55, *p* = 0.04), but no main effect of sense of direction (*F*(1,109) = 0.57, *p* = .45) and no interaction (*F*(4,109) = 1.69, *p* = .16). Since there was a main effect of map condition, but no main effect of sense of direction and no interaction between the two, participants from both SBSOD groups were combined to assess the impact of map condition on RTs in response to different types of landmarks.

**Table 5:** Response times for correct responses to memory test trials, split by landmark type and map condition

Measure	Condition	Landmark type	Mean	SD
Landmark recognition task Response time (correct trials only)	No map	Target	1661	1552
		Incidental	2532	1931
		Distractor	3294	2255
	Simple map study	Target	1724	1520
		Incidental	3348	3918
		Distractor	4736	4850
	Simple map carry	Target	2008	1917
		Incidental	4151	3818
		Distractor	4728	5345
	Complex map carry	Target	1977	2066
		Incidental	3139	2637
		Distractor	4374	3494
	SketchUp carry	Target	1701	1192
		Incidental	4036	3568
		Distractor	3654	2524

A mixed-design ANOVA with the between-subjects factor of condition and the within-subjects factor of landmark type (target or incidental) showed no main effect of map condition (*F*(4,114) = 2.26, *p* = .07), but a significant effect of landmark type (*F*(1,114) = 99.27, *p* < .001), and a significant interaction (*F*(4,114) = 2.70, *p* = .03). The main effect of landmark type indicated that RTs to the incidental landmarks were generally longer than those to the target landmarks. The additional time needed to recall the incidental landmarks likely reflects the weaker memory traces that participants had for these landmarks, given that they were not explicitly pointed out along the route.

The significant interaction between map condition and landmark type indicates that the difference between map conditions depended on the landmark type. Specifically, we can see in Fig. 10 that participants had significantly longer RTs to incidental targets in the simple map carry and SketchUp carry condition relative to the no-map condition (no map vs. simple map carry: *t*(29.84) = 3.30, *p* < .01; no map vs. SketchUp carry: *t*(32.28) = 3.26, *p* < .01). RTs for the simple map study and complex map carry conditions fell somewhere in between and did not differ significantly from either the no-map or simple map carry/SketchUp carry conditions. On the other hand, RTs to target landmarks were relatively stable across map conditions.

### Map completion task

Because different mechanisms are believed to underlie the development of route and landmark knowledge, these two scores will be analyzed separately below.

#### Route score

**Fig. 11:**
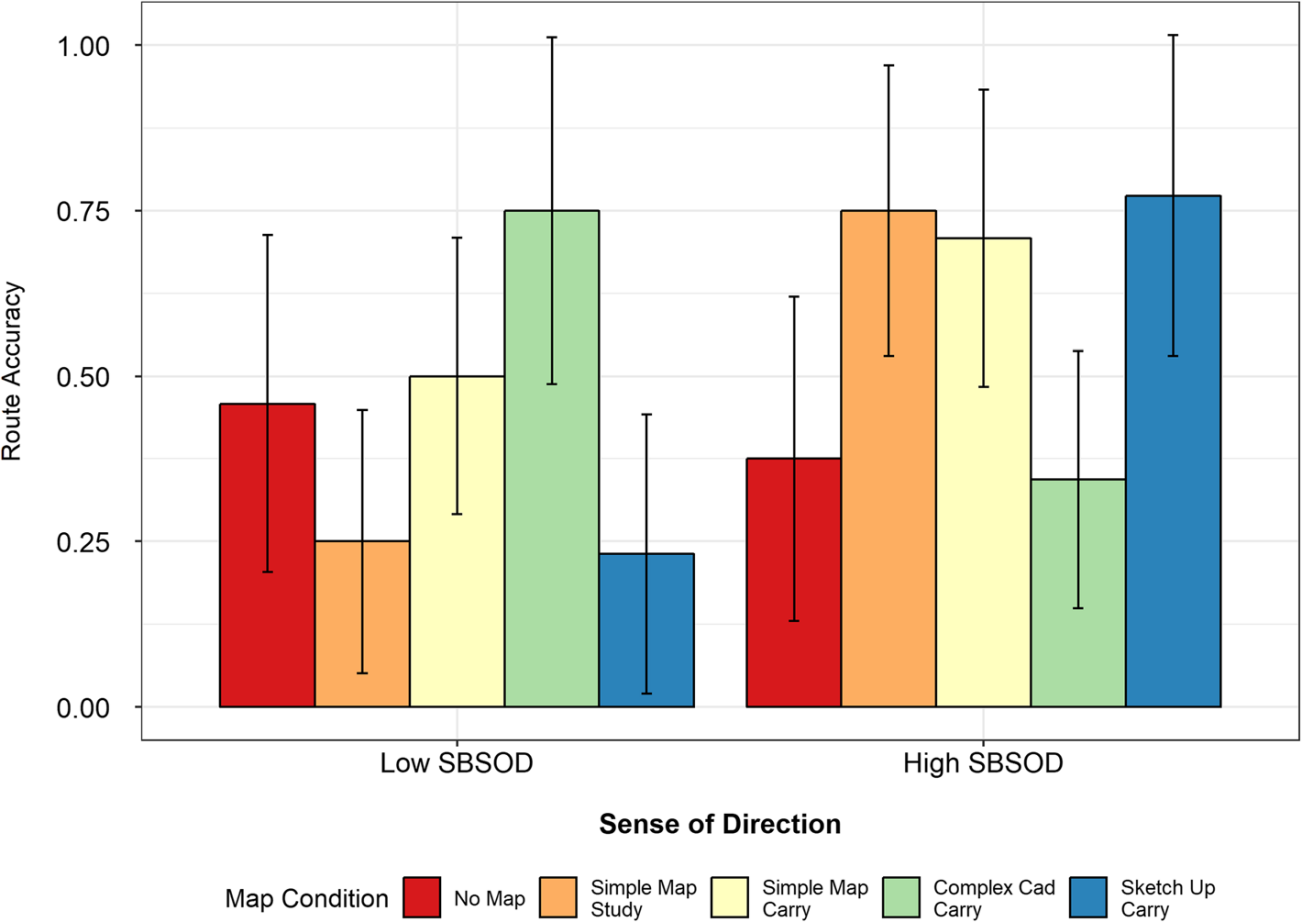
Map completion task route accuracy scores as a function of map condition, split by sense of direction

For the route scores there was a main effect of sense of direction (*F*(1,110) = 4.86, *p* = .03) and a significant interaction between map condition and sense of direction (*F*(4,110) = 5.74, *p* < .01), although again no main effect of map condition was observed (*F*(4,110) = 0.75, *p* = .56). Mean values are shown in Fig. 11.

##### High-SBSOD group

As in the other tasks, participants in the high-SBSOD group had poorer performance in the no-map and complex map carry conditions than they did for the simple map conditions. Within the high-SBSOD group, the no-map condition was significantly worse than the simple map study (*t*(20) = 2.24, *p* = .04) and SketchUp carry (*t*(20.97) = 2.26, *p* = .03) conditions, and marginally worse than the simple map carry condition (*t*(21.83) = 1.97, *p* = .06). Moreover, the complex map carry condition was significantly worse than the simple map study (*t*(20.95) =2.72, *p* = .01), simple map carry (*t*(23.85) = 2.41, *p* = .02) and SketchUp carry (*t*(21.16) = 2.71, *p* = .01) conditions.

##### Low-SBSOD group

As before, the low-SBSOD participants had relatively high performance in the conditions for which the high-SBSOD participants did worst: the no-map condition and the complex map carry condition. However, in a departure from the pattern observed for other tasks, the low-SBSOD participants also performed well in the simple map carry condition. Follow-up *t* tests within the low-SBSOD group found that the complex map carry condition elicited significantly higher scores than either the simple map study (*t*(14.76) = 2.98, *p* = .01) or the SketchUp carry condition (*t*(15.28) = 3.03, *p* = .01). No other pairwise comparisons were significant.

Comparing across the two SBSOD groups showed that they had equivalent performance on the no-map condition (*t*(21.96) = 0.46, *p* = .65) and the simple map carry condition (*t*(21.89) = 1.33, *p* = .19). The participants in the high-SBSOD group had significantly higher accuracy than the participants in the low-SBSOD group on the simple map study (*t*(20.37) = 3.31, *p* < .01) and SketchUp carry conditions (*t*(20.90) = 3.31, *p* < .01), while the participants in the low-SBSOD group had significantly higher accuracy in the complex map carry condition (*t*(14.74) = 2.44, *p* = .03).

#### Landmark score

Just like the route scores, the landmark scores did not show a significant main effect of map condition (*F*(4,110) = 0.58, *p* = .68), but they did show a significant main effect of sense of direction (*F*(1,110) = 10.89, *p* < .01) and a significant interaction (*F*(4,110) = 5.34, *p* < .01).

##### High-SBSOD group

**Fig. 12:**
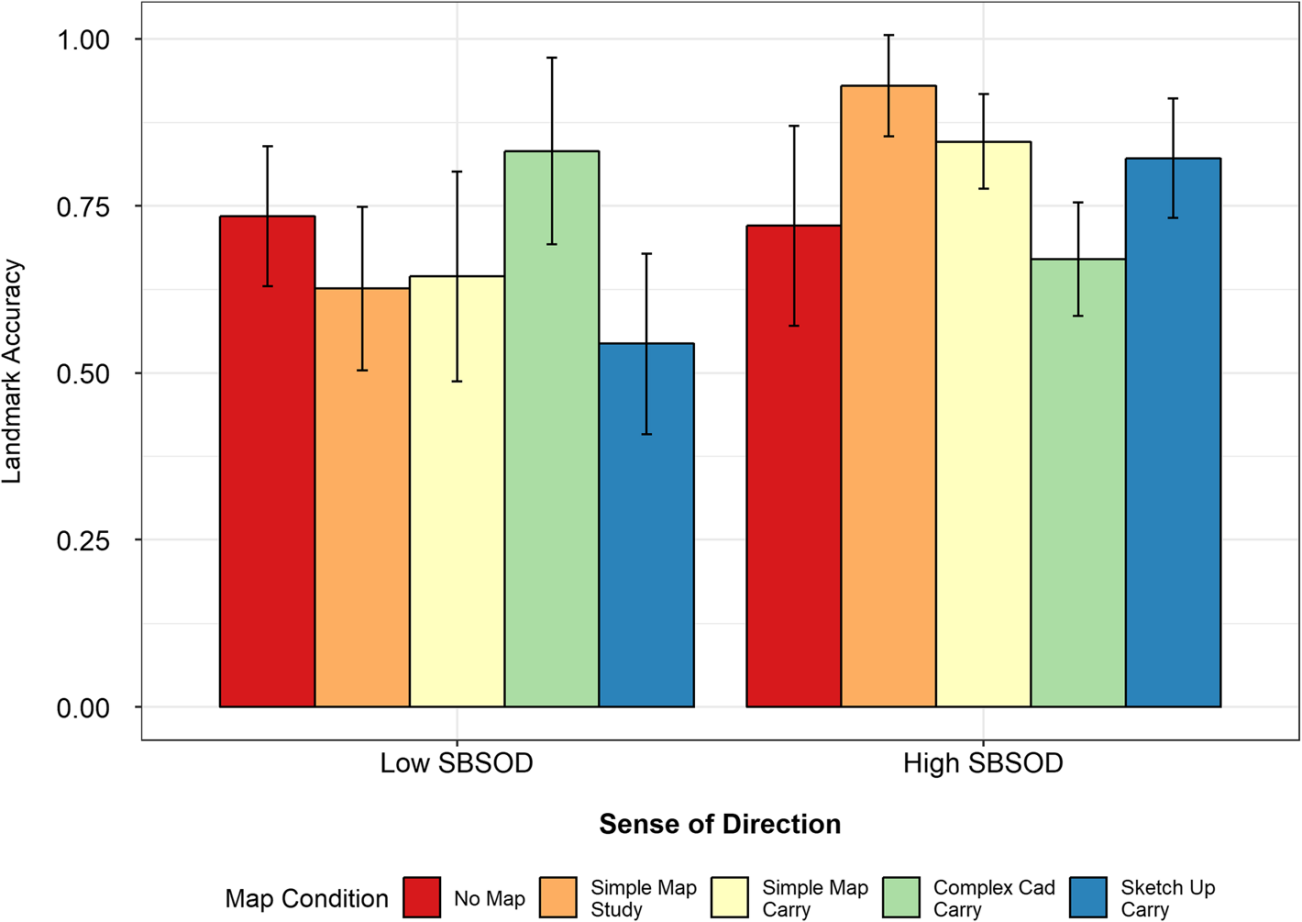
Map completion task landmark accuracy scores as a function of map condition, split by sense of direction

As shown in Fig. 12, the pattern for the landmark scores was very similar to that observed for the other tasks: namely, the no-map and complex map carry conditions elicited the worst performance in the high-SBSOD group and the best performance in the low-SBSOD group. Within the high-SBSOD group, the no-map condition was significantly worse than the simple map study condition (*t*(16.04) = 2.44, *p* = .03), and the complex map condition was significantly worse than the simple map study *(t*(23.54) = 4.47, *p* < .01), simple map carry (*t*(25.98) = 3.13, *p* < .01) and SketchUp carry (*t*(23.46) = 2.40, *p* = .02) conditions.

##### Low-SBSOD group

Within the low-SBSOD group, the no-map condition was significantly better than the SketchUp carry condition (*t*(22.08) = 2.19, *p* = .04). The complex map carry condition was significantly better than the simple map study (*t*(16.56) = 2.17, *p* = .04) and the SketchUp carry condition (*t*(17.35) = 2.91, *p* < .01); no other comparisons reached significance.

When comparing across the two SBSOD groups, we observed that their performance did not differ significantly for the no-map condition (*t*(19.67) = 0.15). However, the high-SBSOD participants outperformed the low-SBSOD participants on the simple map study (*t*(20.53) = 4.14, *p* < .001), simple map carry (*t*(15.28) = 2.30, *p* = 0.04) and SketchUp carry conditions (*t*(20.17) = 3.36, *p* < .01). The low-SBSOD participants numerically outperformed the high-SBSOD participants on the complex map condition, but this difference did not reach significance (*t*(12.34) = 1.94, *p* = .07).

### Noticing inconsistencies between the maps and the building

In the map completion task, participants were asked to note any inconsistencies that they noticed between the map and the building. As we predicted, most participants did not note any differences between the map and the building. When they did notice a difference, it was usually the missing wall in the mezzanine, which was identified by 37 of the 120 participants. The number of participants who noticed the missing wall was not significantly different across conditions (*F*(4,115) = 0.73). Ten participants noticed the missing stairs, five added the cubicles, four participants noted that the section of the catwalk that had been removed, three added the curtain, and two participants noted that the door next to the glove box was blocked. Only one to two participants in each map condition noticed each of these inconsistencies, so there was not enough data to warrant a comparison across conditions.

## Discussion

In the current experiment, we asked whether the provision of certain map types would aid the development of spatial knowledge of a novel environment during a guided route-learning task. We tested the efficacy of three different map types, a simple map, a complex map, and a 3D SketchUp map. In three of the learning conditions, participants studied one of the three maps prior to learning a guided route through an industrial facility, and then carried the maps with them for reference during the route-learning task. In a fourth condition, the participants studied the simple map before learning the guided route but were not allowed to carry the map with them during the route-learning task. All four of these map conditions were compared to a control condition in which participants never received a map of the building before learning the guided route.

Since prior research has found large differences in spatial knowledge acquisition between people with a good or a poor sense of direction (Hegarty et al., 2002; Wolbers & Hegarty, 2010), we used the SBSOD to assess participants’ sense of direction and divided the participants into groups based on a median split on the SBSOD scores. We found that the efficacy of the different map conditions for aiding the development of spatial knowledge was highly dependent on individual differences in sense of direction.

### High-SBSOD group

For people with a good sense of direction, we observed better performance on almost all measures when they were provided with a map—*unless t*hat map was the complex map, in which case the participants performed roughly the same as they did in the no-map condition. This pattern, in which the no-map and complex map conditions produced the worst performance for people with high SBSOD, was especially clear in the pointing task and map completion task (both route and landmark scores) and was less consistent but still numerically present for the shortcut task. This supports our prediction that the extraneous detail on the complex maps would be distracting and would make them more difficult to use. People with a good sense of direction did not benefit from having a more detailed map. This finding replicates Dillemuth (2005), who found that participants with high SBSOD performed better on a route-learning task with a simple versus detailed map.

It is also interesting to note that the high-sense-of-direction group did not show any additional benefits to having the map with them during the route-learning phase (i.e., there were no observable differences between the simple map study and simple map carry conditions), nor did they benefit from the 3D SketchUp map above and beyond the simpler map. The only task on which the map condition did not have an effect for the high-SBSOD group was the landmark recognition task, in which we saw no differences in landmark recognition across the different map conditions. This was the only task that did not require spatial knowledge, since participants were asked whether or not they saw an object in the environment, rather than where in the building it was located.

### Low-SBSOD group

For people with a low-sense-of-direction score, the results were almost reversed from those observed for the high-sense-of-direction group. Namely, the provision of a map tended to produce *lower* performance on almost all tasks—*unless* that map was the complex map, in which case performance was roughly the same as in the no-map condition. This pattern was especially pronounced in the pointing task, shortcut task, and map completion task. Interestingly, in the map completion task, the simple map carry condition also performed on par with the no-map condition, suggesting a slight benefit for the simple map carry for developing this hybrid of route/survey knowledge.

It is notable that whenever there was a main effect of sense of direction for a particular task, the high- and low-SBSOD groups performed equally well on the no-map condition. This was true for the pointing task as well as the for route and landmark scores on the map completion task. In all three cases, when no map was provided, there were no significant differences in performance between the high- and low-SBSOD groups. When given a simple map or a SketchUp map, the high-SBSOD participants performed better on these tasks than they did without a map, while the low-SBSOD participants performed worse (with the exception of the simple map carry condition in the route completion task, as noted above). This indicates that the simpler maps helped the high-SBSOD participants while hindering the spatial learning of the low-SBSOD participants.

When given a complex map, the high- and low-SBSOD groups had equivalent performance on the pointing task, but the low-SBSOD group had significantly higher route scores than the high-SBSOD group on the map completion task. They also had higher landmark scores on the map completion task, although the difference was not statistically significant in that case. This pattern, combined with the pattern observed for the simpler maps, as discussed above, indicates that the low-SBSOD participants may not have attempted to use the complex map, or failed to encode much information about it. This made their performance comparable to the no-map condition, which, for the low-SBSOD participants, was beneficial. The data suggests that the high-SBSOD participants either ignored or were distracted by the complex map. Just like the low-SBSOD participants, their performance was very similar for the no-map and complex map conditions, which suggests that they were not using the complex map. However, the fact that the low-SBSOD participants outperformed them on the map completion task indicates that the complex map may have been distracting for the high-SBSOD participants. In their case, having an overly complex map may be worse than having no map at all. Anecdotally, many participants in both groups complained about the complex map and commented that they did not believe it would be helpful.

Another interesting difference between the high- and low-SBSOD participants emerged for the SketchUp maps. Recall that the SketchUp maps were oriented at a 45° angle relative to the other two maps. When participants completed the pointing task, they were also oriented at a 45° angle relative to the starting point of the route, and this orientation matched the orientation of the SketchUp map. Based on the findings regarding orientation effects in the literature (e.g., Levine et al., 1984; Richardson et al., 1999), we would predict that the SketchUp map would provide the best support for the pointing task, since the studied map corresponded most closely to the demands of the task in this condition. Numerically, the high-SBSOD participants had the highest performance on the pointing task when they were in the SketchUp map condition, while the low-SBSOD participants scored lowest in that condition. The matching orientation may have provided some benefit to the high-SBSOD participants, but it did not benefit the low-SBSOD participants.

Similarly, the SketchUp map produced the worst overall performance on the map completion task for the low-SBSOD group. For this task, the difference in the orientation between the SketchUp map and the test map may have made it more difficult for people with a low sense of direction understand the relationship between these maps. Together, these findings point to the importance of map orientation to the development of spatial knowledge for people with a low sense of direction. These findings are somewhat in contrast with Liben et al. (2008), who found better performance on a map location task with a slightly oblique round map (like our SketchUp condition) relative to a flat map. However, one difference could be that all of our maps were square, whereas Liben et al. (2008) found the biggest benefits for *round* oblique maps, possibly because people were more likely to rotate a round map than a square map to align with their view in space. As such, one recommendation we may give to IAEA inspectors, especially those who may have a low sense of direction, would be to rotate any map that they receive to match their current orientation, as this could help support their spatial knowledge development.

### Overall effects of map condition

The only measure that did not show a significant main effect of sense of direction or a significant interaction between sense of direction and map condition was the landmark recognition task. Importantly, this was the only task that did not require any spatial knowledge on the part of the participants. The participants were shown pictures of objects that they had seen (or not seen) along the learned route, and they were tested on their ability to distinguish between seen and unseen items. They were not asked about the location of any of the tested items. When analyzing the RTs for this task, we observed a significant interaction between the map study condition and the landmark type (target or incidental). Namely, we found that individuals in the simple map carry and SketchUp carry conditions took significantly longer to recognize incidental landmarks than did people in the no-map condition. The average RTs for the simple map study and complex map carry conditions fell directly between the no-map and other conditions. We interpret the longer RTs from participants in the simple map carry and SketchUp carry conditions to indicate greater difficulty in recognizing the incidental landmarks. This suggests that participants who carried easy-to-read maps with them during the route-learning phase paid less attention to the environment around them, most likely because they were devoting more of their attention to the maps. People in the no-map condition had the fastest RTs for this condition, indicating that they had formed the strongest memory traces for the objects in the learning environment. Interestingly, people in the simple map study and complex map carry condition patterned together, with RTs that fell between the no-map condition and the simple map carry and SketchUp carry conditions. The participants in the simple map study condition did not have a map with them during the route, but they may have been trying to recall the map that they had studied previously, drawing some amount of attention away from the environment itself. The results on the other tasks indicate that the participants largely ignored the complex map, but participants did report that they referred to it during the route-learning phase. Eighteen of the 24 participants in the complex map condition (13 in the high-SBSOD group and five in the low-SBSOD group) reported that they referred to the map occasionally, frequently, or very frequently during the route-learning phase, which was quite similar to the self-reports of the participants in other groups (20 participants in the simple map carry and 19 participants in the SketchUp carry conditions reported referring to their map occasionally, frequently, or very frequently). These references drew some attention away from encoding the incidental landmarks in the building, but it seems clear that the participants were less engaged with the complex map than they were with the easier-to-read maps.

One of the main goals of this study was to determine how to support the development of spatial knowledge for an environment without impeding awareness of other factors in the environment. This is important for IAEA safeguards inspectors, who must develop an understanding of a facility’s layout while being alert to safety hazards and anomalies in the environment that could warrant further investigation. Our findings suggest that carrying an easy-to-read map improved spatial learning for participants with a good sense of direction, but it also had a negative impact on participants’ awareness of their environment, regardless of the participants’ sense of direction.

Overall, our findings suggest that different map types have pros and cons for the development of spatial knowledge, depending on the sense of direction of the individual using the map as well as that person’s goals. For individuals with a good sense of direction, the provision of both simple 2D and 3D maps was beneficial to the development of spatial knowledge (especially route and survey knowledge), relative to having no map or an overly complicated and/or difficult-to-read map. These participants benefited from using one of the simple maps, regardless of whether they studied the map before entering the facility or carried the map with them while learning the route. However, the maps could also hinder these participants in some ways. Referring to the simple maps during the route-learning task impacted their encoding of incidental landmarks along the route. Also, there was evidence that these participants were hindered by trying to make sense of the complex maps, impacting their ability to reconstruct their route and the locations of the landmarks on the map completion task.

In contrast, individuals with a poorer sense of direction performed best when they were not provided with a map of the facility, or when they were provided with a complex map (which they proceeded to ignore). This suggests that the participants with a lower sense of direction had to expend additional attentional resources trying to understand the maps and their relationship to the building, and that this effort overshadowed any benefits of receiving the map in the first place. The only task on which receiving a map benefitted the low sense of direction group was the route fill-in portion of the map completion task, which measured a hybrid of route and survey knowledge. Finally, like the participants with a good sense of direction, these participants also had poorer encoding of the incidental landmarks when they were carrying one of the simpler maps, as indicated by significantly longer RTs when they were tested on their memory for the incidental landmarks.

The goal of this work was two-fold. First, we sought to address gaps in the literature on the cognitive science of spatial knowledge development in order to understand the potential impacts of map exposure and map type on the development of spatial knowledge for a novel, indoor environment in a guided navigation task. Second, we sought to use this information to make recommendations to the IAEA regarding empirically supported ways to aid international nuclear safeguards inspectors in their development of spatial knowledge while being led on a guided route through a complex nuclear facility. From our current findings, we would make the following recommendations to the IAEA. First, given the large differences in performance that we observed based on an individual’s sense of direction, we would recommend that inspection team members self-assess their own sense of direction and select roles based on this result. For example, if there is a facility map available, an inspector with a good sense of direction should take on the task of studying the map prior to entering the facility. Inspectors with a poor sense of direction should not attempt to memorize or use a facility map, because even trying to hold a map layout in mind was detrimental to several measures of performance for the low sense of direction group in our study. That being said, we would recommend that even inspectors with a high sense of direction forego consulting a map while being led through the building, given that we did not observe additional benefits for carrying a map relative to studying a map beforehand, but we *did* see a detriment to awareness of the environment when people were actively referring to a map while walking the route. However, when working in teams, it may be beneficial for one inspector (with a good sense of direction) to track the group’s location on a map while other inspectors are tasked with attending to the environment as they move through a facility. When more than one inspector is present, these roles can be divided.

Finally, our results indicate that the simpler the map, the better. We did not find additional benefits for the 3D SketchUp map condition, and indeed found that this map condition may have hindered performance for people with a poorer sense of direction. We also did not find benefits to either group from the complex map (however, it consistently patterned with the no-map condition, suggesting that people were not using it).

Our study is not without caveats. First, the different map conditions employed were somewhat limited, based on the materials that were available for the facility used in the experiment. We also limited ourselves to maps that could easily fit on a piece of paper, to stay in line with the constraints faced by IAEA inspectors in the field (i.e., they are typically unable to bring electronic devices into facilities, so an interactive map was not an option). While the map types used in this study are a realistic representation of the types of maps that are available to safeguards inspectors, there are many other types of maps that might provide better support for the acquisition of route and survey knowledge in complex, indoor environments. Interactive digital maps or schematized maps that emphasize different features of a building (cf. Löwen et al., 2019; Meilinger et al., 2007; Münzer et al., 2012) may outperform any of the map types that we tested in the current study. In domains that are less constrained than nuclear safeguards inspections, these other types of maps may be preferable. Secondly, our participant population was chosen so as to be naïve to the environment; as such, our findings can only answer how well our map conditions help develop spatial knowledge of a completely novel environment. It is possible that receiving a map of a somewhat familiar environment may be helpful to people with a low sense of direction. This may be especially relevant to IAEA inspectors because they will often return to facilities multiple times over the course of several months or years, and thus may have differing levels of existing spatial knowledge on which to build. Thirdly, our participant population did not contain professionally trained inspectors. We might expect that IAEA inspectors, through some combination of training, experience, or both, have developed their own wayfinding or memory strategies to employ during an inspection. Future work could explore any or all of these topics, as each will be important to furthering our understanding of how to best provide IAEA safeguards inspectors with information to aid the efficacy and efficiency of their spatial knowledge development when performing inspections in the field.

### Everyday spatial thinking

In the context of everyday spatial thinking, beyond the realm of safeguards, our results suggest that individual differences in sense of direction are of critical importance in spatial learning tasks. While our high- and low-sense-of-direction groups performed equally well when they were not given maps, their performance diverged when they were given any type of map. The same maps that benefited people with a good sense of direction hindered people with a poor sense of direction.

Some portion of this effect may have been a version of stereotype threat (cf. Massa, Mayer, & Bohon, 2005; Moè & Pazzaglia, 2006). The participants all had a sense of whether or not they were good at navigation and map-reading tasks (after all, the SBSOD, which they completed at the end of the study, is a self-assessment). Participants with a poor sense of direction may have become distracted or frustrated by trying to use the maps, and they may have had an additional cognitive load when trying to relate the map to the building. However, their self-perception of their spatial abilities may also have played a role in their poor performance. When confronted with a map at the beginning of the experiment, they may have assumed that they would do poorly on the task, and then proceeded to do so. When participants with a poor sense of direction did not have to worry about maps or their own map-reading abilities, they performed just as well as the people with a good sense of direction. The impact of people’s beliefs about their own spatial abilities on their spatial learning warrants additional research.

Our findings suggest that self-awareness can help people to maximize their own spatial learning. In real-world activities, people would likely benefit from taking their spatial abilities into account and selecting navigation aids or maps that best support their individual needs. People who know that they have a poor sense of direction can take heart in our findings, and hand their map to a friend or coworker if they find themselves in a real-world spatial learning task. If they do not bother with a map, their acquisition of route and survey knowledge may be just fine.

## Data Availability

The datasets used and/or analyzed during the current study are available from the corresponding author upon request.

## Footnotes

1 Data was collected in two phases approximately 1 year apart. During the second phase of data collection, experimenters were required to enter the building through a different door in order to reach the conference room where the informed consent process and initial map study session took place. When using this alternate entrance to the building, participants walked past an emergency shower that had served as one of the unseen distractor items for the landmark recognition task. Although participants walked past this item prior to beginning the experiment, it was replaced with a different distractor item in the landmark recognition task, just in case participants recognized it and misremembered when they had seen it. Note that the experimental route itself was identical in both phases of data collection.
